# NMR Profiling of European Oak Honeydew Honeys: Saccharide Diversity and Geographical Differentiation

**DOI:** 10.3390/molecules31162913

**Published:** 2026-08-20

**Authors:** Dessislava Gerginova, María del Carmen Seijo Coello, Nikola Jovanović, Marco Ciulu, Svetlana Simova

**Affiliations:** 1 Bulgarian NMR Centre, Institute of Organic Chemistry with Centre of Phytochemistry, Bulgarian Academy of Sciences, Acad. G. Bonchev Str., Bl. 9, 1113 Sofia, Bulgaria; 2 Centre of Competence “Sustainable Utilization of Bio-Resources and Waste of Medicinal and Aromatic Plants for Innovative Bioactive Products” (BIORESOURCES BG), 1000 Sofia, Bulgaria; 3 Department of Plant Biology and Soil Science, Faculty of Sciences, University of Vigo, As Lagoas, 32004 Ourense, Spain; 4 Department of Biology and Ecology, Faculty of Sciences and Mathematics, University of Niš, Višegradska 33, 18000 Niš, Serbia; 5 Department of Biotechnology, University of Verona, Strada Le Grazie 15, 37134 Verona, Italy

**Keywords:** oak honeydew honey, ^13^C NMR, geographical origin, saccharide profile, chemometrics, Strandzha PDO, authentication

## Abstract

Although European oak honeydew honeys have a similar botanical origin, it is unclear if their chemical composition reflects their geographical origin. Sixty samples from Bulgaria, Germany, Greece, Italy, North Macedonia, Romania, Serbia, and Spain were analyzed using semiquantitative ^13^C nuclear magnetic resonance (NMR) spectroscopy. The protected designation of origin (PDO) Strandzha honey was compared to honey from other regions in Bulgaria. The profiles included 25 identified constituents, 21 saccharides, and 16 reproducible unidentified signals. Statistical analysis differentiated country groups through combinations of variables rather than a single marker. A decision tree based on nigerose, fructose, melezitose, panose, sucrose, and one unidentified signal summarized the main differences among the countries. Strandzha honeys exhibited a unique regional profile characterized by higher glucose levels and lower concentrations of several minor saccharides and unidentified signals. Two coordinated saccharide networks revealed that minor sugars fluctuate in linked groups rather than independently. This pattern has not been previously described in European honey. Conventional honeydew-associated indicators varied across the dataset. However, 1-kestose was present in all samples. Nevertheless, its specificity to oak honeydew origin remains unresolved. While extended ^13^C NMR profiling may support geographical characterization, independent validation is required before it can be used for authentication.

## 1. Introduction

Conventionally, honey is classified according to the source of the carbohydrates collected by honeybees: nectar (blossom) honey and honeydew honey. Nectar honey comes from plant nectar, while honeydew honey mainly originates from the excretions of insects that suck the sap from living plant parts, or from secretions of living plant tissues [1]. The formation of honeydew honey involves interactions between the host plant, honeydew-producing insects, honeybees, and the surrounding environment. Variation in insect species, host plants, and environmental conditions can alter honeydew composition, contributing to the substantial chemical diversity of honeydew honeys [2,3].

Although honeydew honey is less extensively studied than nectar honey, it has attracted increasing scientific and commercial interest. It is generally characterized by a dark color, a comparatively high mineral content, and a complex minor metabolite profile. Numerous studies have reported its pronounced antioxidant and antimicrobial properties [4]. Its production varies considerably from year to year due to its dependence on environmental conditions, which contributes to its limited availability and commercial value. Principal European sources include conifers such as *Abies*, *Picea*, and *Pinus*, as well as deciduous and evergreen species of *Quercus*. Coniferous honeydew honey is particularly important in temperate and mountainous regions, while oak honeydew honey is associated with Mediterranean, Balkan, and other southern European forest ecosystems [5,6].

The chemical composition of oak honeydew honey includes compounds associated with the *Quercus* host or honeydew in general. Quercitol has been suggested as an indicator of oak honeydew contribution related to the host, whereas 2,3-butanediol has been more broadly associated with honeydew honey [7,8,9]. However, their consistency across European oak honeydew honeys and their relevance to geographical differentiation remain unclear.

The saccharide profile is one of the most informative compositional features of honeydew honey. As well as fructose and glucose, it comprises numerous disaccharides and trisaccharides originating from the collected honeydew or formed and modified through the activity of honeydew-producing insects and honeybee enzymes [2,10]. Melezitose is commonly regarded as a key saccharide associated with honeydew origin, whereas erlose and raffinose can provide additional evidence to distinguish honeydew from blossom honeys. Nevertheless, saccharide composition alone has not proved sufficient for the consistent differentiation of honeydew honeys according to botanical or geographical origin [11]. As most oak-specific studies have only examined conventional sugars or relatively limited saccharide panels [12,13,14,15], it is unclear whether minor saccharides vary independently or in a coordinated manner that contributes to geographical differentiation.

A detailed analysis of honey saccharides remains challenging due to the close structural relationship of many minor di- and trisaccharides, which requires adequate separation, authentic standards, or complementary structural evidence for reliable identification and quantification [16]. Consequently, incomplete resolution has led to unresolved or combined fractions being reported under a single compound designation, such as “maltose” or “1-kestose”. This limits the ability to make direct comparisons among studies and potentially obscures variations and relationships among individual saccharides [6,17].

NMR spectroscopy combines structural information with quantitative analysis, enabling the simultaneous characterization of sugars and other minor constituents without derivatization or chromatographic separation. While extensive signal overlap in the carbohydrate region may hinder the identification of specific sugars using ^1^H NMR alone, incorporating ^13^C chemical shift data provides substantially greater spectral dispersion, facilitating the differentiation of carbohydrates with similar structures [18]. The lower intrinsic sensitivity of NMR spectroscopy is partly compensated for by the high carbohydrate concentration in honey. The resulting spectra enable the observation of saccharides that may co-elute chromatographically, providing reproducible molecular profiles suitable for chemometric analysis [19].

NMR spectroscopy has been used to characterize and differentiate honeydew honeys botanically, including identifying quercitol as an oak-associated constituent and discriminating honeydew honeys based on their botanical origin [8,20]. Our previous ^13^C NMR study demonstrated the differentiation of Bulgarian and North Macedonian honeys according to their combined botanical and geographical origins. This differentiation was achieved through contributions from both identified and unidentified signals [19]. However, most oak-specific investigations have examined samples from a single country or a limited number of regions. Therefore, it is still unclear whether extended NMR-derived molecular profiles can distinguish between European oak honeydew honeys from different countries and regions.

“Strandzhanski manov med”/“Manov med ot Strandzha” was registered as a Protected Designation of Origin in 2019 [21]. The product’s specifications link it to the oak forests and environmental conditions of the Strandzha Massif, including the interactions among the local vegetation, honeydew-producing insects, and climate. Thus, the designation provides a practical, legally relevant framework for evaluating the regional specificity of Strandzha honey.

This study employed an extended semiquantitative ^13^C nuclear magnetic resonance profiling method to analyze 60 oak honeydew honeys sourced from Bulgaria, Germany, Greece, Italy, North Macedonia, Romania, Serbia, and Spain. The first objective was to determine whether the samples could be differentiated by country of origin based on their complete NMR-derived compositional profiles. A second, more detailed geographical comparison was performed within Bulgaria by evaluating honeys from the Strandzha Protected Designation of Origin (PDO) production area against oak honeydew honeys produced in other Bulgarian regions. Multivariate classification and clustering approaches were used to assess separation at both geographical levels. To explain the chemical basis of these patterns, we examined the dataset for coordinated saccharide relationships, compounds associated with honeydew or oak origin, and reproducible unidentified NMR signals. Thus, this study aimed to determine how individual compounds and their combined variation within the extended molecular profile contribute to the geographical differentiation of European oak honeydew honey.

## 2. Results and Discussion

### 2.1. Geographical Differentiation Among European Countries Based on the Extended ^13^C NMR Profile

**Figure 1:**
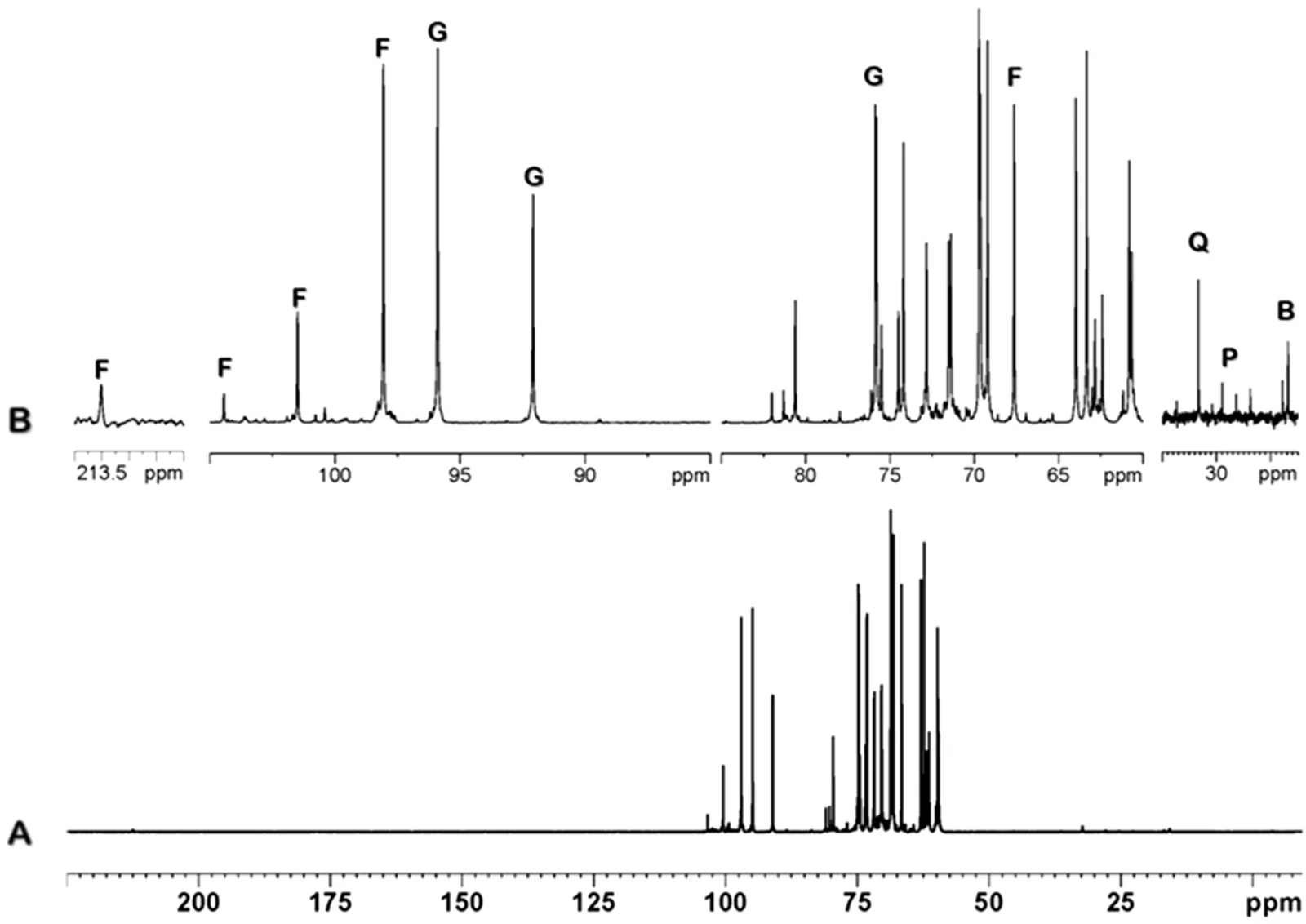
^13^C NMR spectrum of oak honeydew honey sample S17 (PDO): (**A**) Full spectrum. (**B**) The carbonyl, anomeric, saccharide, and aliphatic regions of the spectrum, annotated with glucose (G) and fructose (F) in the carbonyl and anomeric regions, as well as the two signals used for quantification in the saccharide region. The aliphatic region shows the signals of quercitol (Q), proline (P), and 2,3-butanediol (B).

Using the semiquantitative ^13^C NMR procedure, 25 constituents were determined in 60 oak honeydew honeys. These constituents included two monosaccharides, 13 disaccharides, six trisaccharides, quercitol, proline, *meso*-2,3-butanediol, and *rac*-2,3-butanediol. Sixteen reproducible yet currently unidentified signals were evaluated and retained as part of the extended molecular profile. Figure 1 shows a representative ^13^C NMR spectrum with selected assignments. Figure S1 shows expanded saccharide regions of selected spectra illustrating the resonances used to profile the minor saccharides. Table S1 provides details on the samples’ declared geographical origin and classification, and Table S2 presents the mean concentrations and ranges of the assigned constituents and unidentified signals for each country.

**Figure 2:**
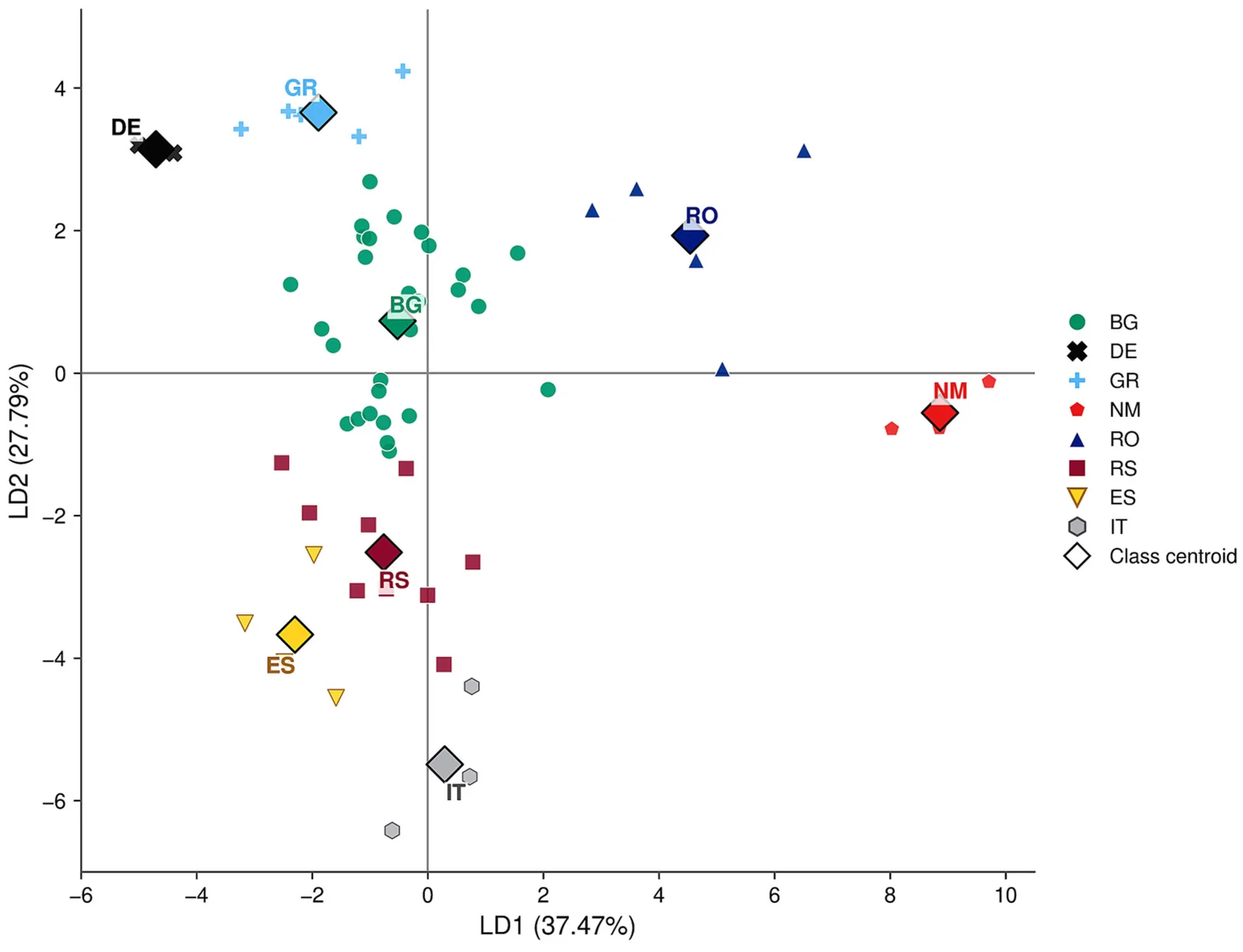
LDA score plot of the 60 European oak honeydew honeys, grouped by their country of origin.

Within this quercitol-positive background, the 60 oak honeydew honeys exhibited similar overall ^13^C NMR profiles. To determine if compositional variation within this shared profile contained geographical origin information, an eight-class linear discriminant analysis (LDA) model was constructed using honeys collected from eight European countries. The optimized LDA model retained sixteen variables: glucose, fructose, kojibiose, nigerose, α,α-trehalose, α,β-trehalose, trehalulose, *rac*-2,3-butanediol, the summed contribution of the unidentified signals U1–U16, and seven of these signals considered separately (U1, U3, U6, U11, U13, U14, and U16). The model generated seven discriminant functions. The first three accounted for 37.47%, 27.79%, and 16.68% of the total discriminant variance, respectively. Together, they represent 81.94% of the country-related separation. The score plot of the first two discriminant functions revealed distinct, albeit partially overlapping, geographical distributions (Figure 2).

The North Macedonian and Romanian samples were predominantly located on the positive side of LD1, while the German, Spanish, and Greek honeys showed mainly negative scores. Kojibiose and the unidentified signals U6, U16, and U11 loaded positively on LD1. Meanwhile, nigerose and the summed U1–U16 fraction were associated with the negative direction of LD1. LD2 further differentiated the Greek and German honeys from the Italian, Spanish, and Serbian samples. Negative LD2 scores corresponded mainly to trehalulose, glucose, α,α-trehalose, fructose, and U3. The Spanish group was characterized by positive LD3 scores, to which U13, nigerose, and U14 contributed most strongly. In contrast, the Bulgarian samples occupied a broad central region, reflecting substantial within-group variability and the absence of a single dominant discriminant direction. Their position exemplifies a broader feature of the model: country-level separation arises from different combinations of assigned compounds and unidentified spectral signals across several discriminant functions rather than from a single variable common to all geographical groups. This structure should not be interpreted as a direct effect of national boundaries. Rather, the country classes likely integrate variation in local *Quercus* populations, honeydew-producing insect communities, environmental conditions, and other regional factors that influence honeydew formation and subsequent honey maturation. Thus, the LDA resolved composite geographical profiles rather than chemically uniform national signatures. The complete loadings of all sixteen retained variables across the seven discriminant functions are provided in Table S3.

The model assigned all 60 samples to their declared countries when evaluated using the original dataset. Leave-one-out cross-validation correctly classified 53 of the 60 honeys, corresponding to an accuracy of 88.3% (95% confidence interval: 77.8–94.2%; Table S4). This included 28 of the 29 Bulgarian samples, all German, Greek and North Macedonian samples, and four of the five Romanian honeys. The remaining errors occurred predominantly among the Serbian, Spanish and Italian groups. The difference between the original and cross-validated results indicates that the apparent separation was not equally stable across all geographical classes. Previous studies indicate that the geographical discrimination of honeydew honeys depends strongly on both the analytical profile and the geographical scale examined. Microscopic characteristics distinguished Greek pine from fir honeydew honeys but did not differentiate pine honeys according to production region [22]. In contrast, volatile profiling classified Greek *Q. ilex* honeydew honeys according to regional provenance with an accuracy of approximately 80% [23], whereas elemental profiling achieved 91.3% correct classification of bracatinga honeydew honeys from three Brazilian production regions [24]. The geographical classification of honeydew honeys from different countries has been examined less frequently. Front-face fluorescence spectroscopy could not distinguish fir honeydew honeys from a geographically restricted area spanning Germany and Switzerland [25]. In our previous ^13^C NMR study of Bulgarian and North Macedonian honeys, 37 out of 38 samples were correctly classified across five combined botanical and geographical groups. However, the smaller geographical scale and different class structure preclude direct numerical comparison [19]. Attempts have also been made at more complex, multicountry classification, but it remains dependent on the analytical profile and is not uniform across geographical classes. Mostoles et al. used multiclass PLS-DA with physicochemical, spectrophotometric, and LC-MS/MS polyphenolic data from honeys sourced from eight countries [26]. Classification performance varied among countries and analytical datasets. In this context, the present cross-validated accuracy of 88.3% is notable because it was obtained through a multiclass comparison of oak honeydew honeys from eight European countries using a single ^13^C NMR-derived molecular profile dominated by saccharides and minor metabolites.

**Figure 3:**
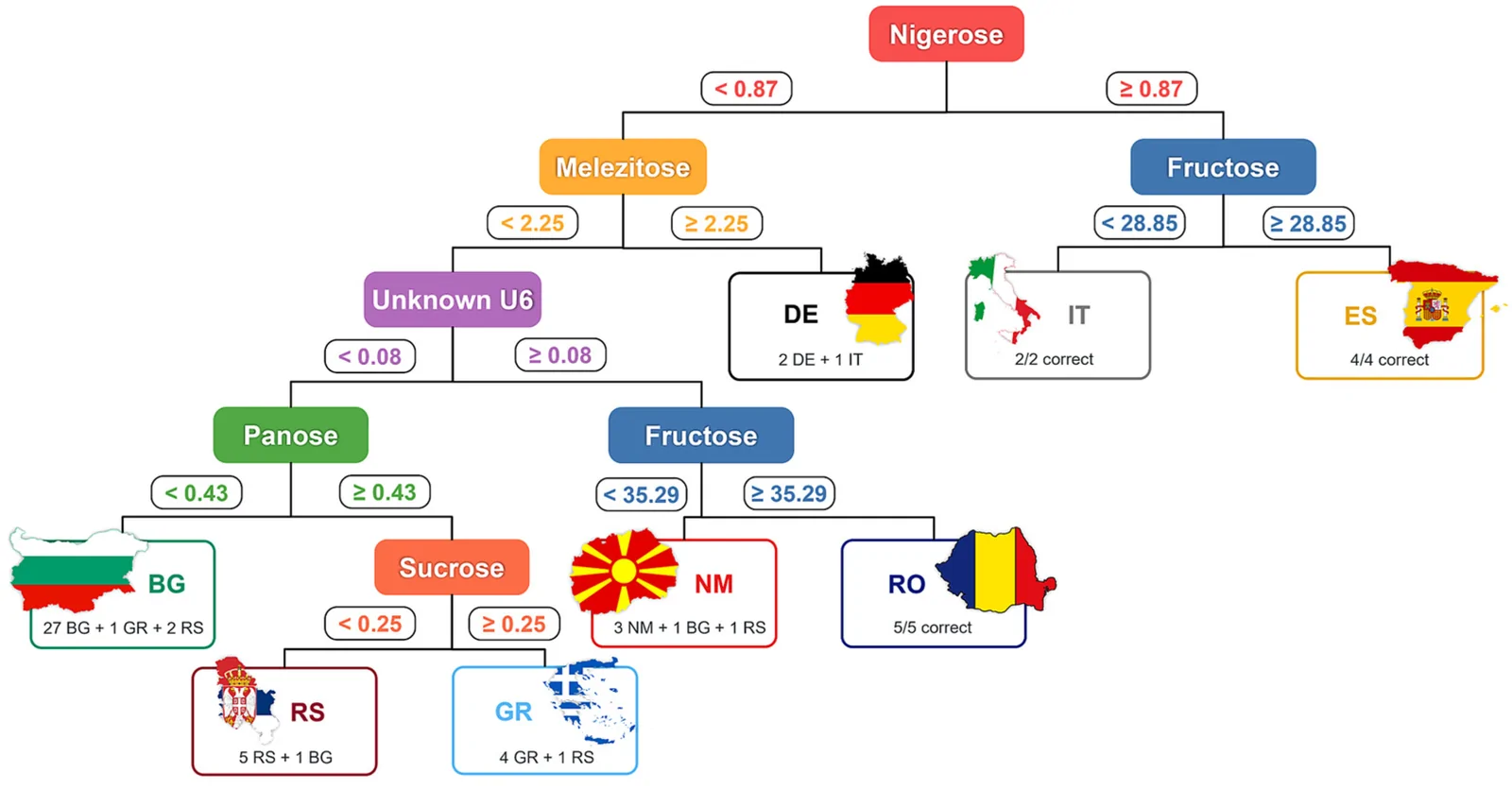
Classification tree of European oak honeydew honeys according to their country of origin. Thresholds are expressed in g/100 g.

To determine whether the multidimensional differentiation identified by LDA could be represented by a smaller number of chemically interpretable concentration thresholds, we constructed a decision-tree model (Figure 3).

The primary division was provided by nigerose at 0.87 g/100 g. Samples above this threshold were subsequently separated by fructose concentration, producing a pure Spanish terminal node and a node containing two of the three Italian honeys. Among samples with lower nigerose concentrations, a melezitose threshold of 2.25 g/100 g separated DE1, DE2, and IT1—the only three honeys that exceeded this concentration. The remaining samples were successively divided according to U6, panose, fructose, and sucrose concentrations. The Romanian and Spanish samples formed country-specific terminal nodes. In contrast, the branches dominated by Bulgarian, Serbian, Greek, and North Macedonian honeys also included samples from other countries.

The tree correctly assigned 52 of the 60 samples to their declared countries, corresponding to an apparent accuracy of 86.7% (95% confidence interval: 75.8–93.1%). Most misclassifications involved Serbian and Bulgarian honeys, which is consistent with their partial overlap in the LDA score plot. In practical terms, the resulting thresholds could offer preliminary information about the potential origin of a newly analyzed sample. This would eliminate the need for a complete multivariate workflow or characterization of every constituent in its molecular profile. The proposed origin could then be evaluated using the full statistical model. The presence of U6 in the tree and the retention of several unidentified signals in the LDA model indicate that some geographical information resides in unassigned spectral features that require further characterization.

The Bulgarian subset provided an additional level of geographical resolution because it included honeys from the Strandzha region, which is associated with a Protected Designation of Origin (PDO), as well as from several other Bulgarian production areas. Therefore, it was examined separately to determine if the NMR profile retained discriminatory information at a finer, legally relevant regional scale.

### 2.2. Regional Differentiation of Strandzha PDO and Other Bulgarian Oak Honeydew Honeys

**Figure 4:**
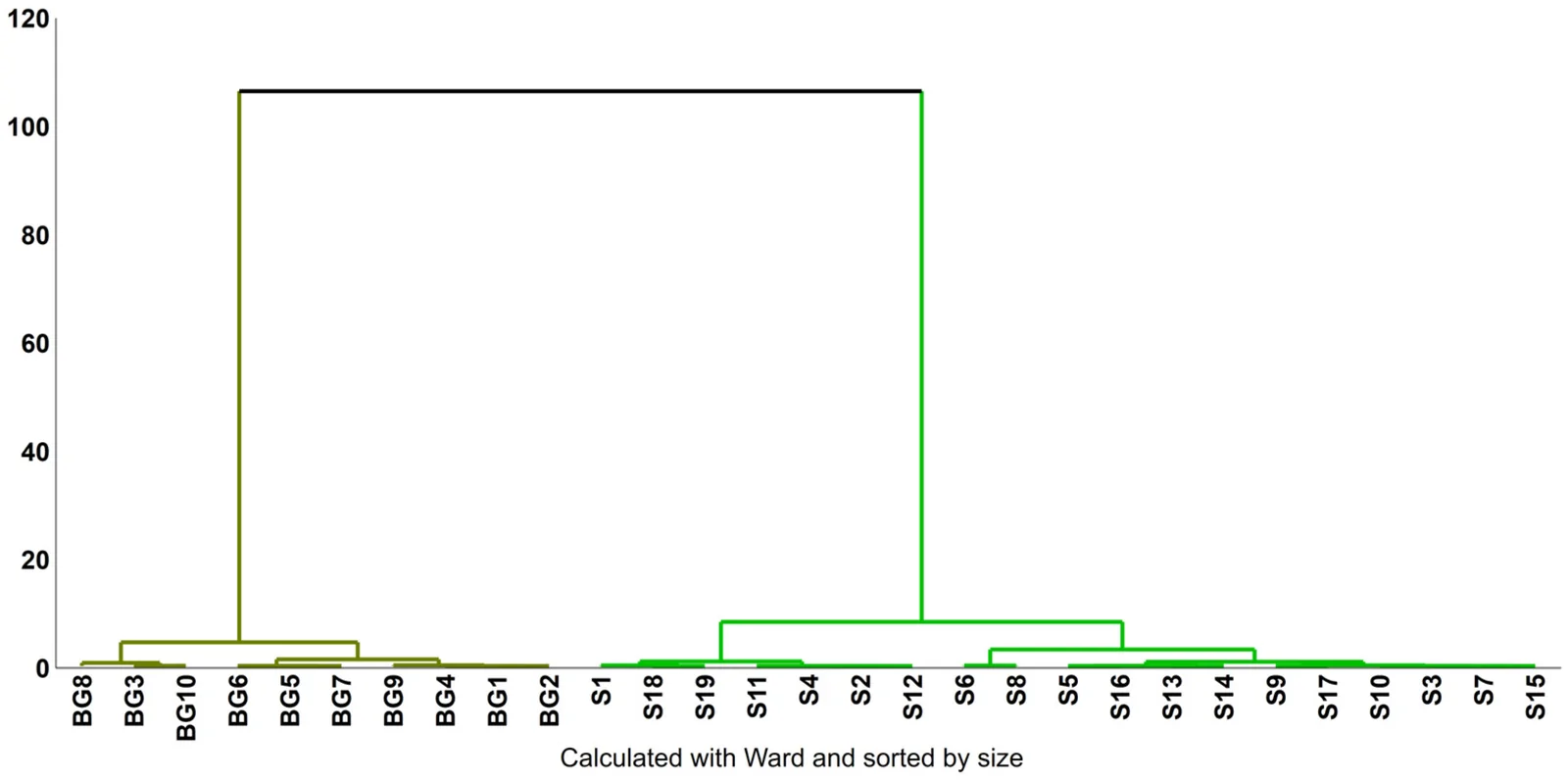
HCA dendrogram of the 29 Bulgarian oak honeydew honeys based on their complete NMR profiles.

The Bulgarian subset included 29 oak honeydew honeys, of which 19 were from the Strandzha Protected Designation of Origin (PDO) production area and 10 were from other regions in Bulgaria. First, hierarchical cluster analysis was applied to determine if this regional distinction was reflected in the overall molecular profiles, independent of geographical class labels. This analysis included all quantified constituents and reproducible unidentified signals, and it was performed using Ward’s clustering method. The resulting dendrogram contained two main clusters that corresponded exactly to the Strandzha and Other_BG groups (Figure 4). This showed that the regional structure was evident in the unsupervised organization of the molecular profiles and did not arise solely from subsequent supervised modeling.

OPLS-DA was subsequently applied to characterize the regional separation and identify the variables contributing most strongly to the distinction between the two groups. Following model optimization, 12 variables with VIP values above 0.9 were retained: glucose, gentiobiose, leucrose, maltose, nigerose, trehalulose, raffinose, the summed contribution of the unidentified signals U1–U16, and the individual signals U1, U10, U11 and U14. Quercitol, melezitose and 2,3-butanediol, compounds previously associated with the characterization of oak or honeydew honeys, were not retained among the influential regional variables. The final model comprised one predictive and three orthogonal components. It explained 84.0% of the variation in the NMR data (R^2^X = 0.840) and 78.0% of the class-related variation (R^2^Y = 0.780), with a cross-validated predictive ability of Q^2^ = 0.377. To assess the robustness of the supervised separation and the possibility of model overfitting, several internal validation diagnostics were examined. Cross-validated predictions correctly assigned all 29 samples (Table S5). The model was significant according to CV-ANOVA (*p* = 0.042), and ROC analysis yielded an area under the curve of 1.00 for both classes (Figure S2). The original R^2^ and Q^2^ values also exceeded those obtained after 200 random permutations of the class labels (Figure S3), providing additional evidence that the observed regional separation was not generated by random class assignment.

**Figure 5:**
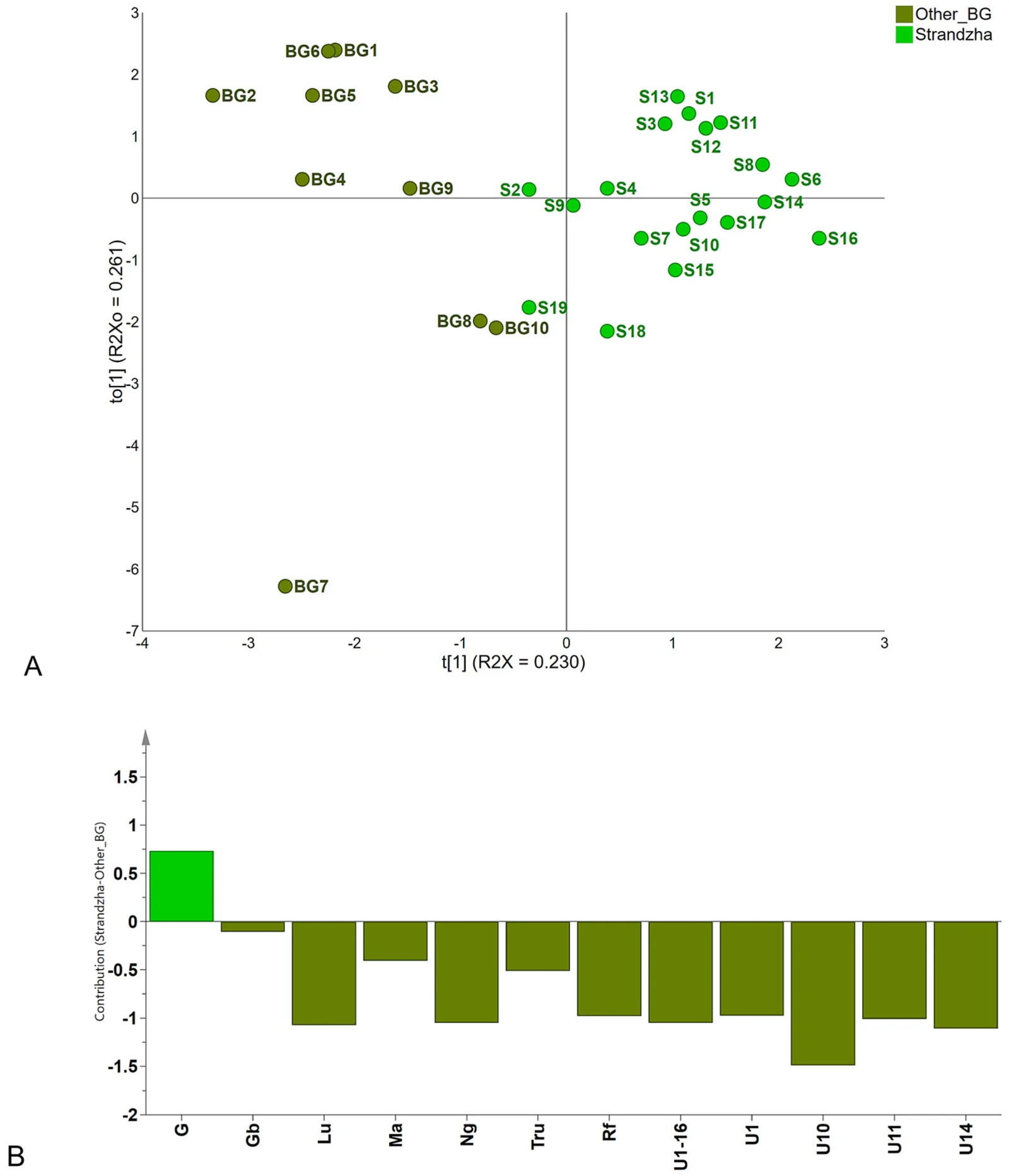
OPLS-DA differentiation of Strandzha PDO honeys and honeys from other Bulgarian regions: (**A**) score plot; (**B**) contribution plot of the retained variables.

The score plot showed complete separation between the Strandzha and Other_BG samples (Figure 5A). One sample, BG7 from the Elenski Balkan region, was markedly displaced from the remaining Other_BG honeys, predominantly along the orthogonal component. Nevertheless, it remained clearly on the Other_BG side of the predictive component and was correctly classified. Therefore, its position reflected pronounced compositional variability within the Other_BG group rather than uncertainty in its regional assignment.

To examine the chemical basis of the regional separation, we evaluated the contributions of the retained variables and their mean concentrations in the two groups (Figure 5B). Glucose contributed most strongly to the Strandzha class, while the other selected variables were associated with the Other_BG class. The mean glucose concentration was 34.10 g/100 g in Strandzha honey and 31.03 g/100 g in samples from other Bulgarian regions. However, the Strandzha group had lower mean concentrations of leucrose (0.19 vs. 0.32 g/100 g), maltose (0.91 vs. 1.12 g/100 g), nigerose (0.51 vs. 0.63 g/100 g), trehalulose (1.04 vs. 1.26 g/100 g), and raffinose (0.29 vs. 0.49 g/100 g). Gentiobiose, the summed contribution of the unidentified signals U1–U16, and the individual signals U1, U10, U11, and U14 were also associated with Other_BG. U10 showed the largest standardized contribution. Thus, the regional distinction reflected a combined shift in the relative balance of glucose and several minor saccharides, as well as structurally unidentified NMR signals, rather than an isolated difference in a single constituent. BG7 further illustrated the multivariable nature of the regional distinction. The sample had comparatively high glucose, lower raffinose, and summed U1–U16 values, which were more characteristic of the Strandzha profile. However, it also had elevated leucrose and maltose, which were associated with Other_BG. This mixed compositional pattern was consistent with its displacement along the orthogonal component; however, the broader configuration of variables preserved its assignment to the Other_BG class.

### 2.3. Chemical Basis of Geographical Differentiation

Country- and regional-scale models showed that geographical information was distributed across combinations of NMR detectable constituents rather than concentrated in a single compound. To interpret the chemical basis of these multivariate patterns, we considered the quantitative dataset from three complementary perspectives: the composition and coordinated relationships of the saccharides; the diagnostic consistency of compounds previously associated with honeydew or oak origin; and the contribution of structurally unidentified NMR signals.

#### 2.3.1. Saccharide Composition and Coordinated Relationships

The saccharide dataset included two monosaccharides, thirteen disaccharides, and six trisaccharides. Their concentrations and frequencies of occurrence are summarized in Table 1. Fructose and glucose dominated the quantitative profile. The minor saccharides turanose, maltulose, isomaltose, trehalulose, and maltose occurred at the highest mean concentrations. The trisaccharides were generally less abundant and showed greater differences in frequency and distribution. The complete saccharide correlation matrix is provided in Table S6. This compositional hierarchy raised the question of whether the individual saccharides varied independently or formed coordinated profiles whose relative expression contributed to geographical differentiation.

Although there was comparatively limited differentiation among countries, glucose contributed directly to the finer regional separation within the Bulgarian subset. Therefore, it was examined first within the broader saccharide profile. Glucose ranged from 23.21 to 40.63 g/100 g with an overall mean of 32.06 g/100 g. Country means occupied a considerably narrower interval of 29.51–33.93 g/100 g, with the highest means observed in Italian and Bulgarian honeys and the lowest mean concentration observed in the German group. The remaining country means were concentrated between 30.03 and 31.35 g/100 g, with substantial within-country variability, particularly among the Serbian samples, which covered almost the entire individual range. The overall glucose concentration exceeded most previously reported oak-specific values. Spanish *Quercus* honeys generally contained 24.10–27.22 g/100 g; Croatian *Q. frainetto* honeydew honey contained 28.70 g/100 g; and Turkish oak honeys contained approximately 21.73–28.33 g/100 g [5,7,12,13,14,15,27,28,29]. Values reported for honeydew honeys of unspecified botanical origin were more varied. The Serbian and Croatian means of approximately 31.00–31.91 g/100 g were close to the present overall concentration [6,30]. In contrast, the Romanian, Czech, and North American datasets showed lower values of approximately 26.08–28.20 g/100 g [16,31,32,33]. In contrast, the value of 36.80 g/100 g reported for North Macedonian honeydew honey was the only one considered here that exceeded the present overall mean [6]. Thus, the present glucose levels were higher than those in most published oak-specific datasets, yet they remained within the broader compositional range reported for honeydew honeys. Glucose exhibited inverse relationships with several sugars, including raffinose (Spearman ρ = −0.77), isomaltose (ρ = −0.45), fructose (ρ = −0.37), and panose (ρ = −0.34). Pascual-Maté et al. similarly reported a strong inverse correlation between glucose and isomaltose in Spanish honeys of different botanical origins (r = −0.82), suggesting that this association may extend to honeys of diverse origins. However, in contrast, glucose and fructose were strongly positively related in their dataset (r = 0.80) [28]. The inverse relationship observed here indicates that the association between the two major monosaccharides is inconsistent across honey datasets. These correlations alone cannot determine whether the observed relationships originate in the collected honeydew or emerge subsequently during honey maturation.

Fructose exhibited greater variation among country means than glucose. Its concentrations ranged from 24.36 to 38.96 g/100 g with an overall mean of 32.82 g/100 g, and country means ranged from 27.95 g/100 g in Italian honeys to 37.58 g/100 g in Romanian honeys. The Bulgarian, Greek, and North Macedonian samples had nearly identical means of 33.55–33.60 g/100 g. The German, Serbian, and Spanish groups had means ranging from 30.04 to 31.20 g/100 g. The low Italian mean contrasted with the high glucose concentration in the same samples, indicating a marked imbalance between the two primary sugars. The overall fructose mean was close to those reported for two larger Spanish *Quercus* datasets (32.58–32.80 g/100 g), whereas mean concentrations across Spanish oak-specific datasets ranged more broadly from 32.00 to approximately 38.30 g/100 g [5,7,13,27]. Similar values have been reported for Croatian *Q. frainetto* honeydew honey [15]. Turkish oak datasets range from 31.62 to 43.28 g/100 g [12,14,29]. Honeydew honeys not assigned to a specific oak source exhibited similar variation. The mean fructose concentration was approximately 31.80–33.80 g/100 g for Croatian, North American, Swiss, and Czech honeydew honeys, which encompasses the present overall mean [6,16,32,34]. Higher values of 35.52, 36.35, and 37.24 g/100 g were reported for Romanian, Montenegrin, and Serbian honeydew honeys, respectively [30,31,35]. Fructose was inversely associated with a broad range of minor saccharides. The strongest relationships were observed with gentiobiose, 1-kestose, panose, and nigerose (Spearman ρ = −0.51 to −0.43), while weaker negative associations were observed with turanose, leucrose, maltotriose, α,β-trehalose, α,α-trehalose, maltulose, erlose, and trehalulose. Raffinose was the only quantified saccharide that was positively related to fructose (ρ = 0.47), while no significant relationship was observed with melezitose. A comparable inverse fructose-panose association was reported in Spanish honeys of different botanical origins (r = −0.72) [28]. This broad pattern of correlations indicates that variation in fructose was linked to coordinated changes across the minor saccharide profile rather than to fructose-containing disaccharides alone.

Despite contrasting geographical patterns of fructose and glucose, their combined concentration ranged from 54.11 to 71.30 g/100 g with an overall mean of 64.88 g/100 g, exceeding the 45 g/100 g minimum established for honeydew honey and honeydew-blossom blends [1]. This value closely matched the mean of 64.5 g/100 g reported for Croatian *Quercus frainetto* honeydew honey, the mean of 64.37 g/100 g found in a Turkish *Quercus* series, and the mean of approximately 65 g/100 g reported for Turkish *Quercus robur* honey [12,14,15]. The F/G ratio exhibited a clearer geographical structure than the combined fructose and glucose concentration. Country means ranged from 0.83 in Italian honey to 0.97 in Spanish honey and 1.21 in Romanian honey, while Greek and North Macedonian samples showed intermediate means of 1.11–1.12. Ratios below 1 occurred in some Bulgarian and Serbian honeys. All three Italian samples contained more glucose than fructose. These values were generally lower than those reported for oak honeydew honey, ranging from approximately 1.20 to 1.46 in Spanish *Quercus* datasets, averaging 1.26 in Croatian *Q. frainetto* honeydew honey, and extending from approximately 1.12 to 1.99 in Turkish oak honey [5,7,12,13,14,15,27,28,29]. The low ratios in the Italian and Spanish groups reflect a distinct monosaccharide balance. The present Spanish samples differ from previously published Spanish *Quercus* profiles. Overall, the major-sugar data suggest that geographical variation is expressed more through the balance between fructose and glucose than through total fructose-plus-glucose content and more through the relationship between the two monosaccharides and the minor-saccharide profile.

Not all sugars that are routinely quantified participated in broader coordinated relationships. Sucrose and maltose are commonly considered in the assessment of honey quality and authenticity, but their associations within the present saccharide profile were comparatively limited. Sucrose concentrations remained low throughout the dataset, quantified in 58 of the 60 samples at levels up to 1.04 g/100 g. The overall mean was 0.29 g/100 g, with country-specific means ranging from 0.15 g/100 g in Romanian honey to 0.65 g/100 g in Greek honey. All concentrations were well below the regulatory maximum of 5 g/100 g, and the overall mean was consistent with the 0.17–0.37 g/100 g reported for larger Spanish oak honeydew datasets [1]. It was also close to the 0.47 g/100 g found in Turkish oak honey [13,14,28]. Nevertheless, higher concentrations have been reported for individual Spanish *Q. ilex* honeys, Croatian *Q. frainetto* honeydew honey, and Romanian oak honey [7,15,36]. The low concentrations observed here are consistent with extensive sucrose hydrolysis during honey maturation. However, low sucrose concentration alone does not exclude supplementary feeding or adulteration.

Maltose was present in all samples at concentrations ranging from 0.35 to 2.57 g/100 g, with an overall mean of 1.03 g/100 g. The highest country means were observed in the Greek and Serbian groups at 1.79 and 1.45 g/100 g, respectively. Meanwhile, the North Macedonian and Spanish honeys contained the lowest amounts. Published oak-specific values varied widely. For example, Turkish *Q. robur* honey contained only 0.19 g/100 g of maltose, while 1.50 and 1.90 g/100 g were reported for Romanian *Quercus* honey and Croatian *Q. frainetto* honeydew honey, respectively [12,15,36]. Spanish oak honeys showed particularly broad variation: approximately 0.70–2.25 g/100 g in individual *Quercus pyrenaica* and *Quercus ilex* honeys, and 4.32–4.90 g/100 g in two larger *Quercus* datasets [5,7,13,28]. Therefore, the present Spanish mean of 0.59 g/100 g was below previously published Spanish values. However, direct comparison among studies is limited because maltose is often reported as a collective fraction containing other reducing disaccharides [6]. Elevated maltose levels may also result from the addition of maltose-rich sugar syrups [28]. However, maltose concentration alone cannot confirm adulteration. In the current saccharide profile, sucrose was mainly associated with 1-kestose, while maltose was associated with maltotriose. Both sucrose and maltose contributed to the classification models: sucrose to a lower branch of the decision tree, and maltose to the regional OPLS-DA model. However, neither showed broad relationships with the coordinated minor-saccharide profile.

A more coherent pattern emerged among several minor disaccharides, beginning with the coordinated variation in turanose, maltulose, and trehalulose. Turanose was present in all 60 samples, with concentrations ranging from 1.06 to 2.70 g/100 g and an overall mean of 1.82 g/100 g. Spanish and Italian honeys had the highest country means, at 2.48 and 2.20 g/100 g, respectively. German and Romanian honeys had the lowest amounts. The Spanish mean was consistent with the reported range of 2.08–3.10 g/100 g for Spanish oak honeydew honey [5,7,13]. Similar concentrations of 2.00–2.30 g/100 g have been reported for Czech and Romanian honeydew honeys of unspecified botanical origin [31,32,33]. However, a much lower value of 0.80 g/100 g was found in Montenegrin honeydew honey [35]. Turanose was found to correlate positively with maltulose (Spearman ρ = 0.67) and weakly with trehalulose (ρ = 0.40), suggesting a coordinated pattern among these three minor disaccharides. The formation of turanose during honeybee processing through α-glucosidase-mediated transglucosylation has been demonstrated experimentally, although the present data cannot distinguish turanose that was already present in the collected honeydew from turanose that was formed subsequently during honey maturation [37].

The geographical patterns of maltulose and trehalulose were similar to those of turanose. Maltulose was quantified in 58 samples at concentrations up to 5.49 g/100 g, with an overall mean of 2.11 g/100 g, the highest among the disaccharides. Trehalulose was present in all samples at concentrations between 0.54 and 3.58 g/100 g, with an overall mean of 1.35 g/100 g. Both compounds were particularly abundant in Spanish and Italian honeys. Country-specific means were 4.68 and 3.15 g/100 g for maltulose, and 2.19 and 2.74 g/100 g for trehalulose. These values exceeded those reported for Spanish *Quercus ilex* honey: 1.88 g/100 g of maltulose and 0.82 g/100 g of trehalulose [7]. Maltulose and trehalulose were positively correlated (Spearman ρ = 0.47). Together with turanose, the sum of their country means was 9.35 g/100 g in Spanish samples and 8.09 g/100 g in Italian samples, compared to 3.59–5.60 g/100 g in the other groups. This coordinated enrichment occurred in the two groups with the lowest fructose concentrations and F/G ratios. Thus, the distinctive monosaccharide balance of the Spanish and Italian honeys was accompanied by marked enrichment in turanose, maltulose, and trehalulose. While these associations do not establish direct precursor-product relationships, they demonstrate that geographical differences involved coordinated shifts across the saccharide profile rather than the independent variation in individual sugars.

The coordinated pattern was not limited to glucose–fructose disaccharides, but also included glucose–glucose disaccharides. Isomaltose was quantified in all samples, ranging from 0.71 to 4.02 g/100 g with an overall mean of 1.62 g/100 g. The highest country means were found in Spanish and Italian honeys at 2.81 and 2.69 g/100 g, respectively. The North Macedonian group followed with a mean of 2.02 g/100 g. Romanian samples showed the lowest mean, at 1.14 g/100 g. The overall concentration was comparable to the 1.46–1.75 g/100 g reported for Spanish *Quercus* honeys [13,28]. However, the Spanish mean in this study exceeded the 1.11 g/100 g calculated for two extensively characterized *Quercus ilex* samples [7]. A markedly lower concentration of 0.49 g/100 g was reported for Montenegrin honeydew honey of unspecified botanical origin [35]. Isomaltose was found to correlate most strongly with trehalulose (Spearman ρ = 0.75), followed by maltulose (ρ = 0.55) and turanose (ρ = 0.53). Pascual-Maté et al. likewise reported strong positive relationships between isomaltose and gentiobiose and panose (Pearson r = 0.82 and 0.84, respectively), as well as the inverse relationship with glucose discussed above [28]. In the present dataset, however, both associations were substantially weaker, with Spearman ρ values of 0.27 for gentiobiose and 0.25 for panose; neither association remained significant after Benjamini–Hochberg correction. Isomaltose was more closely related to trehalulose, maltulose, and turanose instead. Thus, the coordinated variation in isomaltose was evident in both datasets, but its closest saccharide partners differed.

Kojibiose was quantified in all samples at concentrations ranging from 0.43 to 1.41 g/100 g, with an overall mean of 0.87 g/100 g. The highest country means were found in Spanish and North Macedonian honeys at 1.31 and 1.21 g/100 g, respectively. The Italian group followed with a mean of 1.07 g/100 g. The current Spanish mean is lower than the 1.90 g/100 g reported for two Spanish *Quercus ilex* honeys [7]. The comparatively high North Macedonian value differs from the predominantly Spanish-Italian enrichment observed for turanose, maltulose, trehalulose, and isomaltose. However, it reproduces the higher kojibiose content found in North Macedonian oak honeydew honey than in Bulgarian oak honeydew honey in our previous independent dataset [19]. Kojibiose correlated most strongly with trehalulose and turanose (ρ = 0.68 and 0.60), followed by isomaltose and maltulose (ρ = 0.54 and 0.47). Kojibiose also contributed to the country-level LDA, particularly to the position of the North Macedonian group. Thus, both glucose–glucose disaccharides participated in a broader coordinated network, though their geographical distributions were not identical.

The coordinated relationships observed for turanose, maltulose, trehalulose, isomaltose, and kojibiose were further extended by nigerose and α,β-trehalose. Both were quantified in all 60 samples, with nigerose ranging from 0.22 to 1.20 g/100 g and α,β-trehalose ranging from 0.01 to 0.71 g/100 g. Their respective overall means were 0.57 g/100 g and 0.36 g/100 g. Spanish honeys had the highest mean concentrations of both compounds at 1.13 g/100 g and 0.59 g/100 g, respectively. Italian honeys had the second highest mean concentration of nigerose at 0.80 g/100 g. These values are close to the mean concentrations of 1.16 g/100 g of nigerose and 0.44 g/100 g of α,β-trehalose reported for Spanish *Quercus ilex* honey [7]. The two compounds exhibited a strong correlation (Spearman ρ = 0.91) and positive relationships with all five previously discussed disaccharides. The coefficients were ρ = 0.61–0.83 for nigerose and ρ = 0.45–0.83 for α,β-trehalose. The inclusion of nigerose in this pattern was reflected in its selection as the root split of the decision tree at 0.87 g/100 g, capturing the broader minor disaccharide enrichment of the Spanish group and part of the Italian group rather than identifying Spanish origin independently. Reports of higher nigerose concentrations in chestnut honey than in honeydew honey further suggest that its relevance in the present dataset should not be equated with specificity to honeydew origin [38].

The remaining disaccharides were not as uniformly integrated into the coordinated pattern. Isomaltulose was quantified in 44 samples at concentrations of up to 0.92 g/100 g, with an overall mean of 0.34 g/100 g, and it was most abundant in Italian honeys (0.80 g/100 g). The North Macedonian and Spanish honeys followed with concentrations of 0.51 and 0.43 g/100 g, respectively. These concentrations exceeded the 0.24 g/100 g reported for Spanish *Quercus ilex* honey [7]. The relationships were relatively limited, with the strongest being observed with trehalulose (Spearman ρ = 0.48). Leucrose was present in 59 samples at concentrations up to 2.70 g/100 g, with an overall mean of 0.36 g/100 g. The high German mean of 1.44 g/100 g was largely driven by one sample. In contrast, the Spanish group had a mean of 0.83 g/100 g. Leucrose was moderately related to isomaltose, trehalulose, nigerose, kojibiose, and α,β-trehalose (ρ = 0.45–0.55). It was also retained in the regional OPLS-DA and was associated with the Other_BG class.

Gentiobiose was quantified in only 31 samples at concentrations of up to 0.46 g/100 g, with an overall mean of 0.10 g/100 g; its highest country mean was found in Spanish honey (0.28 g/100 g), followed by Italian and German honey (0.19 and 0.17 g/100 g, respectively). Gentiobiose was not detected in North Macedonian or Romanian honey. The means for the Spanish, Italian, and German samples all exceeded the 0.05 g/100 g reported for Spanish *Q. ilex* honey [7]. Gentiobiose’s strongest association was an inverse relationship with fructose (ρ = −0.51). Despite its limited occurrence, gentiobiose contributed to the regional model.

α,α-Trehalose differed markedly from α,β-trehalose in both distribution and correlation structure. α,α-Trehalose was quantified in 46 samples at concentrations of up to 4.58 g/100 g. Its mean concentration of 0.49 g/100 g substantially exceeded the median concentration of 0.12 g/100 g, reflecting the influence of a few highly enriched samples. The highest country means were found in Serbian and German honeys at 1.77 and 1.67 g/100 g, respectively. The Italian group followed with 0.93 g/100 g. However, 0.65 g/100 g was reported for Spanish *Q. ilex* honey [7]. Unlike α,β-trehalose, α,α-trehalose was most closely associated with panose, melezitose, and erlose (ρ = 0.45, 0.41, and 0.34, respectively).

Taken together, the most consistent coordinated relationships were found with turanose, maltulose, trehalulose, isomaltose, kojibiose, nigerose, and α,β-trehalose. Leucrose was moderately associated with five of these compounds but did not show comparable relationships with turanose or maltulose. Isomaltulose showed only limited coupling, principally with trehalulose. Therefore, these two disaccharides were not included as integral members of the coordinated pattern. Jia et al. previously identified a coordinated core comprising turanose, maltulose, kojibiose, and isomaltose in Chinese monofloral nectar honeys of diverse botanical origins [39]. They associated this core with linked transformations during honey ripening. The recurrence of this core in European oak honeydew honey demonstrates that this association is not limited to a specific botanical source, honey type, or geographical location. To our knowledge, this study is the first to provide evidence of this coordinated core in European honey, extending it to a seven-disaccharide pattern through the additional participation of trehalulose, nigerose, and α,β-trehalose. Thus, the occurrence of the pattern itself does not appear to be geographically specific, whereas the relative abundance of its individual members may carry geographical information.

The trisaccharides exhibited significantly greater variation in frequency and distribution than the disaccharides, and their associations were less uniformly organized. Several were related to α,α-trehalose, while selective relationships with fructose, glucose, sucrose, and maltose were also observed.

Among the trisaccharides, melezitose and 1-kestose showed the clearest contrast in occurrence, distribution, and correlation structure. Melezitose was quantified in only 33 samples and exhibited highly asymmetric distribution, ranging from 0 to 3.74 g/100 g. The mean concentration was 0.30 g/100 g, whereas the median was just 0.04 g/100 g. Only five samples contained more than 1 g/100 g, and 27 of the 60 quercitol-positive honeys contained no quantifiable melezitose. Published data likewise show substantial variation in melezitose concentration. Oak-specific values range from approximately 0.10 g/100 g in Spanish *Quercus pyrenaica* honeydew honey to 0.31 g/100 g in Turkish *Quercus* honey and approximately 0.90 g/100 g in Spanish *Quercus ilex* honey [5,14]. Tedesco et al. reported even wider variation among three Italian honeydew samples containing 0.87, 10.21, and 15.52 g/100 g of melezitose, respectively, yielding a mean of 8.82 g/100 g [38]. These results illustrate that high mean concentrations may be driven by a few strongly enriched samples rather than representing a consistent feature of honeydew honey. Melezitose was positively associated with α,α-trehalose (Spearman ρ = 0.41), linking it to the more variably expressed oligosaccharide relationships centered on this disaccharide.

In contrast, 1-kestose was quantified in all 60 samples at concentrations ranging from 0.12 to 1.17 g/100 g. The mean and median values were nearly identical at 0.49 g/100 g. Country means were also relatively similar, with Bulgarian, Spanish, Greek, and North Macedonian honeys containing approximately 0.51–0.53 g/100 g, moderately exceeding the mean of 0.36 g/100 g calculated for two Spanish *Quercus ilex* honeys [7]. 1-Kestose was positively associated with sucrose and inversely related to fructose, linking its variation to sucrose transformation and the major-sugar balance. Its universal occurrence and comparatively symmetrical distribution therefore distinguished it from melezitose and the other more variably expressed trisaccharides.

Panose showed a more heterogeneous distribution and was quantified in 54 samples at concentrations of up to 3.87 g/100 g, with an overall mean of 0.45 g/100 g. The highest country mean, 1.13 g/100 g, was observed in Serbian honeys, although it was influenced by a limited number of enriched samples. The Spanish mean of 0.37 g/100 g was approximately twice the mean of 0.19 g/100 g reported for two *Q. ilex* honeys [7]. Panose was positively associated with α,α-trehalose (Spearman ρ = 0.45) and inversely related to fructose and glucose, placing it within the more variably expressed set of relationships centered on α,α-trehalose. It was also selected as a splitting variable in one of the lower branches of the decision tree.

Maltotriose was detected less consistently, occurring in 35 samples at concentrations up to 1.12 g/100 g, with an asymmetric distribution reflected by a mean of 0.22 g/100 g and a median of 0.04 g/100 g; it occurred most frequently in the Serbian and Greek groups, though it was not quantified in North Macedonian honeys. The Spanish mean of 0.08 g/100 g was close to the mean of 0.12 g/100 g calculated for the two Spanish *Q. ilex* honeys [7]. However, the overall mean was comparable to the approximately 0.30 g/100 g found in Czech honeydew honey [32]. Maltotriose was significantly associated with maltose and showed a weaker positive tendency with α,α-trehalose. It was also inversely related to fructose. Therefore, panose and maltotriose linked the more variable trisaccharide profile to the broader monosaccharide profile, while maltotriose showed only a tendency toward the pattern centered on α,α-trehalose. However, their relationships were less extensive than those within the coordinated disaccharide pattern.

Erlose was quantified in 58 samples at concentrations of up to 2.54 g/100 g, with an overall mean of 0.45 g/100 g. The highest country means occurred in Greek and Serbian honeys, which is consistent with the distribution of panose and maltotriose. In contrast, Spanish and North Macedonian honeys had comparatively low concentrations. The current mean for Spanish honey is 0.18 g/100 g, which is substantially lower than the reported mean of approximately 0.84 g/100 g for Spanish *Q. ilex* honey [7]. Erlose was positively associated with α,α-trehalose (ρ = 0.34) and inversely related to fructose, connecting these variably expressed oligosaccharides further. Erlose exceeded melezitose in 49 of the 60 samples, indicating a predominantly erlose-rich trisaccharide profile. However, erlose may arise not only from the collected honeydew, but also from honeybee-mediated transformation of sucrose by α-glucosidase [37]. Therefore, its distribution may reflect both the original honeydew source and subsequent honey maturation rather than a single, source-related process.

Raffinose was quantified in all 60 samples at concentrations ranging from 0.04 to 0.90 g/100 g, with an overall mean of 0.35 g/100 g. The highest country mean was found in North Macedonian honeys. The Spanish group contained only 0.20 g/100 g. Both the overall mean and the mean for the Romanian group matched the 0.35 g/100 g reported for Romanian honeydew honey [31]. However, this value exceeds the 0.04–0.15 g/100 g found in Spanish and Croatian oak honeys [7,13,15]. Nevertheless, comparisons among studies should be made cautiously because some chromatographic methods may not completely resolve raffinose from structurally related trisaccharide isomers, including theanderose. Raffinose exhibited a unique relationship with the major sugar balance, negatively correlating with glucose (Spearman ρ = −0.77) and positively with fructose (ρ = 0.47). Raffinose was also retained in the regional OPLS-DA and was associated with the Other_BG class. Its universal occurrence and contrasting relationships with the two major monosaccharides distinguish it from the more variably expressed trisaccharides. Its specificity as a honeydew-associated indicator is discussed separately below.

The associations of melezitose, panose, and erlose with α,α-trehalose defined a second, more variably expressed oligosaccharide pattern centered on this disaccharide, with maltotriose showing a weaker tendency in the same direction. Although these compounds are commonly quantified together in honey studies, and erlose and melezitose have both been associated with honeydew formation, this coordinated pattern of associations has not previously been described.

Taken together, the saccharides did not vary independently, but rather, they were organized into two distinct patterns of expression. The first pattern was more coherent and involved turanose, maltulose, trehalulose, isomaltose, kojibiose, nigerose, and α,β-trehalose. The second pattern was less cohesive and centered on α,α-trehalose. Geographical differentiation was primarily evident in the balance of fructose and glucose and the relative abundance of these coordinated saccharide groups, rather than in the total fructose–glucose concentration or any individual oligosaccharide. The comparatively symmetrical and universal occurrence of 1-kestose among the trisaccharides contrasted with the episodic enrichment of melezitose. This provides the basis for reassessing their relative diagnostic value in the following section.

#### 2.3.2. Reassessment of Proposed Honeydew- and Oak-Associated Indicators

Several minor constituents have been proposed as indicators of honeydew or oak origin. However, they provide different types of evidence, ranging from direct links to *Quercus* tissues to effects associated with honeydew-producing insects, honeybee processing, and honey maturation. Therefore, the present assessment focused primarily on the consistency with which each compound occurred across the 60 European oak honeydew honeys from the eight countries examined, rather than on its contribution to geographical discrimination. A combined oak honeydew authenticity profile would be expected to recur across samples, even if its concentration varies geographically.

Quercitol was considered first because it is a natural constituent of oak tissues and provides the most direct chemical link to the *Quercus* host. It has also been previously proposed as an indicator of *Quercus* honeydew honey. De la Fuente et al. detected quercitol in Spanish *Quercus ilex* honey, and *Simova* et al. found quercitol in all fifteen oak honeydew honeys examined, but not in six blossom honeys or two fir and spruce honeydew honeys [7,8]. These findings support interpreting quercitol as a *Quercus*-associated component rather than a general honeydew honey constituent. However, samples were selected using a quercitol threshold of 0.22 g/100 g, so the presence in all 60 samples does not demonstrate its universality among European oak honeydew honeys. Rather, the criterion provided a common, host-related basis for evaluating the remaining proposed indicators across the eight countries examined.

Next, we examined melezitose because it is the trisaccharide most closely associated with honeydew rather than blossom origin. Honeydew-producing insects synthesize melezitose from sucrose in ingested plant sap. While melezitose remains the most reliable indicator of honeydew contribution, it cannot be used to identify the botanical or geographical origin of honeydew honey [11,16]. In the current oak dataset, melezitose was detected in only 33 out of 60 samples. No quantifiable signal was observed in the remaining 27 samples. Therefore, the presence of a high concentration of melezitose may suggest a honeydew origin, but its absence does not rule out an oak honeydew origin. The performance of the dataset differed markedly among the countries examined. Both German samples were melezitose-rich, but the small sample size precludes broader geographical conclusions. Melezitose was not quantified in the North Macedonian honeys and was practically absent from the Greek and Romanian samples. It was present in 17 of the 29 Bulgarian samples and seven of the nine Serbian samples, though generally at low concentrations. Only a few samples from Italy, Serbia, and Bulgaria showed pronounced enrichment. Five samples exceeded 1 g/100 g: DE1 and DE2 from the Thuringian Forest at 2.52 and 3.74 g/100 g, respectively; IT1 from Trentino at 3.40 g/100 g; RS9 from the Blace area at 1.98 g/100 g; and BG7 from the Elenski Balkan region at 1.38 g/100 g. These honeys originated from four widely separated, forested, mountainous or upland regions. Since honeydew sugar composition may depend more strongly on the honeydew-producing insect than on the host tree, and since temperature and relative humidity also affect melezitose production, the observed enrichment may reflect localized insect–host systems under favorable microclimatic conditions [2,3]. However, the small number of enriched samples and the lack of comparable, site-level information for the remaining honeys prevent definitive generalization. Therefore, while pronounced enrichment may characterize particular honeydew-producing systems, melezitose was not a consistent component of oak honeydew honey in Europe across the eight countries examined.

Next, we considered erlose and raffinose, as both have been suggested as indicators of honeydew presence. However, the evidence supporting their diagnostic value is less consistent than that for melezitose [11,28]. Erlose was quantified in 58 of the 60 samples, but it has also been found in blossom honeys. It may also be formed from sucrose during honeybee processing [28,37,38]. The two erlose-negative samples (S12 from Bulgaria and IT2 from Italy) lacked maltotriose, melezitose, and panose but retained 1-kestose and raffinose. Therefore, the absence of erlose in these samples was part of a broader depletion of less consistently detected trisaccharides rather than an isolated compositional feature. The widespread occurrence of erlose indicates that it is a common component of the oak honeydew honeys but not as a specific indicator of honeydew or oak origin. Raffinose was even more consistent, being quantified in all 60 samples from all eight countries examined. This distribution suggests that it may be a common component of European oak honeydew honey. However, its diagnostic value is limited because it has also been detected in blossom honeys and reported absent from some honeydew honeys [11,28,38]. Thus, its occurrence was consistent within the current European oak dataset. However, this does not establish its specificity to oak origin or its presence in all honeydew honeys. Nevertheless, the widespread occurrence observed here warrants further evaluation in independent, geographically diverse oak honeydew datasets.

We examined 2,3-butanediol as another compound previously associated with honeydew origin. Soria et al. reported positive relationships between the two stereoisomeric forms and the estimated honeydew proportion, as well as several physicochemical characteristics, in Spanish honeys. However, the compound also contributed to differentiation between production areas [9]. Therefore, it is considered a honeydew-associated constituent rather than a specific indicator of *Quercus* origin [40]. In the present dataset, at least one form was quantified in all Bulgarian, Spanish, Greek, North Macedonian, and Romanian samples, as well as six of the nine Serbian honeys. In contrast, it was present in only one of the three Italian samples and practically absent from the German group. Within-country variation was also evident. All four Spanish honeys tested positive, despite originating from Asturias, Galicia, and Andalusia. Among the Italian samples, it was present only in the Calabrian honey and absent from the Apulian and Trentino honeys. Similarly, three Serbian samples from different production areas tested negative. These contrasts are more consistent with variation among localized *Quercus*–honeydew–insect systems and their associated environmental conditions than with simple national or latitudinal patterns. However, the small sizes of several groups and the lack of standardized ecological information prevent a more specific interpretation. The distribution of 2,3-butanediol was largely independent of that of melezitose. It was present in many samples with no quantifiable melezitose. Conversely, the melezitose-rich German honeys contained little to no 2,3-butanediol, and their combined concentrations were not significantly correlated (Spearman ρ = −0.15, *p* = 0.245). Therefore, these two compounds appear to reflect different aspects of localized honeydew-producing systems rather than being interchangeable indicators. Overall, 2,3-butanediol was prevalent but not ubiquitous in European oak honeydew honey. Its consistent presence in several country groups suggests it may be valuable for particular local profiles. However, its limited presence in German and Italian samples prevents it from being used as a pan-European indicator without further validation in larger datasets, including blossom and non-oak honeydew honey.

Unlike the previous compounds, proline is primarily produced by honeybees, so it was examined separately. It is mainly used as an indicator of honey maturation and total amino acid content, rather than as an indicator of a specific plant source [4]. Iglesias et al. reported a significantly higher mean proline concentration in honeydew than in floral honeys (90.46 versus 67.15 mg/100 g of dry matter) [27]. In the present dataset, proline was quantified in all 60 samples at 0.04–0.18 g/100 g, with an overall mean equivalent to 817 mg/kg. Country means occupied a relatively narrow range of 0.06–0.10 g/100 g and overlapped extensively. This concentration was comparable to published values for oak honeydew honey [14,41]. However, Mitlianga et al. found no significant difference between oak honeydew and oak honeydew-nectar honeys, with respective means of 965.6 and 1095.7 mg/kg [41]. Proline is a consistent component of oak honeys and is compatible with normal honey maturation. Due to its predominantly bee-derived origin and limited specificity, however, proline is only useful as a supporting quality parameter, not as an independent indicator of honeydew or oak origin.

Against this background, 1-kestose emerged as the primary additional candidate identified in the present dataset. It was quantified in all 60 samples from eight countries. Similar concentrations were reported in two Spanish *Quercus ilex* honeys [7]. Unlike the variable detection of melezitose, 2,3-butanediol, and erlose, the consistent presence of 1-kestose across a broad geographic range suggests that it is a consistent component of the European oak honeydew profile. However, this evidence is unidirectional; the consistent presence of 1-kestose in oak honeydew honey does not prove that the honey is from oak honeydew. The compound has also been detected in blossom honeys. Additionally, a chromatographic peak tentatively assigned to 1-kestose was reported in fir and spruce honeydew honey, though it was not fully separated from nigerose and stachyose [17]. Conversely, 1-kestose was absent from the six non-oak Nothofagus honeydew honeys examined by Astwood et al. [42]. These observations suggest that 1-kestose’s occurrence may vary among honeydew-producing systems, but they do not establish its specificity for *Quercus*-derived honeydew. Thus, while 1-kestose exhibited consistent results within the current European oak honeydew dataset, its diagnostic specificity remains undetermined. Before it can be applied as a stand-alone authenticity marker, it must be validated in independent, geographically diverse oak honeydew datasets, together with blossom and non-oak honeydew controls analyzed using the same semiquantitative nuclear magnetic resonance method. For now, it is best considered a candidate indicator within a combined compositional profile, alongside quercitol and the broader saccharide pattern.

Overall, the proposed indicators varied substantially in terms of consistency and diagnostic interpretation. Quercitol provided the clearest evidence related to the host. In contrast, melezitose and 2,3-butanediol reflected more variable contributions from localized honeydew-producing systems. Erlose, raffinose, and proline were common constituents, but they lacked specificity. The complete occurrence of 1-kestose made it the strongest additional candidate identified, though its specificity to oak honeydew remains unestablished. Therefore, no single assigned compound captured the samples’ full compositional diversity. This is consistent with the dependence of the multivariate models on combinations of quantified constituents and unidentified NMR signals. The latter are discussed in the following section.

#### 2.3.3. Unidentified Components and the Need for Extended Profiling

The repeated contribution of unidentified NMR signals to the country- and regional-level models revealed that chemically unresolved variation was an important component of the geographical profiles. Further correlation analysis showed that U1–U16 did not behave as a coherent group. The strongest relationship was observed between U3 and U4 (Spearman ρ = 0.67). The remaining associations were weaker and defined several partly independent patterns. Individual signals also exhibited distinct relationships with the assigned compounds. For example, U11 was strongly associated with isomaltose and trehalulose (ρ = 0.85 and 0.81, respectively), while U13 correlated with maltulose, kojibiose, trehalulose, and nigerose (ρ = 0.58–0.63). U3 was positively related to maltose (ρ = 0.53), but negatively related to maltulose and trehalulose (ρ = −0.40 and −0.39). U8 correlated negatively with raffinose and fructose (ρ = −0.53 and −0.45), and positively with glucose (ρ = 0.37). U1 and U10 were most closely associated with proline, and U5 and U7 showed positive relationships with 1-kestose. The absence of uniformly strong intercorrelations, together with these distinct associations with the assigned constituents, strongly suggests that U1–U16 reflect multiple, chemically distinct constituents, arguing against a single-compound origin. However, the lack of structural identification limits the chemical interpretation of their contribution and currently prevents their use as targeted authenticity markers. More detailed characterization using complementary two-dimensional nuclear magnetic resonance experiments, isolation procedures, mass spectrometry, and comparison with authentic standards is therefore required. Identifying these constituents could clarify the chemical basis of the observed geographical differentiation and expand the molecular profile available for authenticating European oak honeydew honey.

Therefore, the chemical basis of the observed geographical differentiation was multicomponent and profile-based. It arose from the combined variation in assigned constituents and reproducible unidentified signals, rather than from any single conventional indicator.

### 2.4. Limitations

Several limitations should be considered when interpreting the present results. First, the country groups were markedly imbalanced, with some represented by only two to four samples. As the effects of country, region, and provider could not be separated; the observed country-level patterns should be considered exploratory rather than validated national profiles. Although formal evaluation of the LDA assumptions was precluded by the small class sizes, the pooled within-class covariance matrix was full rank and the condition index was 12.3, indicating no severe numerical instability. Second, all multivariate patterns and classification estimates were obtained from the available datasets without external validation. Therefore, the LDA and OPLS-DA performance may be optimistic, and the exact HCA structure and the exploratory decision-tree thresholds require confirmation in independent samples. Additionally, the regional comparison was limited to the available Bulgarian dataset and should be validated using additional Strandzha and non-Strandzha honeys. Detailed information on the storage and thermal history of the samples prior to their receipt by the laboratory was unavailable for all samples. Conclusions based on quercitol threshold sampling are restricted to the oak honeydew profile represented by samples meeting the ≥0.22 g/100 g criterion. The structural identity of several influential NMR signals remains unresolved. Thus, larger, more balanced and independent datasets are required, along with blossom and non-oak honeydew controls, before the proposed profiles and indicators can be used for authentication purposes.

## 3. Materials and Methods

### 3.1. Honey Samples and Selection Criteria

An initial set of 75 honeys, all of which were declared as oak honeydew honey and originating from nine European countries, was examined. The declared botanical and geographical origins were based on information provided by beekeepers, commercial suppliers, or product documentation. Because of its established association with Quercus-derived honeydew [8], quercitol was used as the principal selection criterion. Samples containing less than 0.22 g/100 g quercitol were excluded. Details of the 15 excluded samples and their quercitol concentrations are provided in Table S7. Additionally, no characteristic resonances of kynurenic acid, a compound associated with chestnut nectar honey [43], were observed in the ^1^H NMR spectra. The final dataset comprised 60 samples from Bulgaria (*n* = 29), Germany (*n* = 2), Greece (*n* = 5), Italy (*n* = 3), North Macedonia (*n* = 3), Romania (*n* = 5), Serbia (*n* = 9), and Spain (*n* = 4). The Bulgarian subset included 19 samples originating from the Strandzha PDO production area and 10 samples from other Bulgarian regions. The declared geographical origin and classification of each sample are provided in Table S1. The samples were analyzed shortly after they were acquired. Prior to analysis, they were stored in closed containers, protected from light and kept at room temperature. Where available, collection years are reported in Table S1. No characteristic resonances attributable to 5-hydroxymethylfurfural (5-HMF) were observed in the ^1^H NMR spectra. Country-specific mean concentrations and ranges of the assigned constituents and unidentified signals are provided in Table S2.

### 3.2. Sample Preparation

A total of 320 mg of each homogenized honey sample was mixed with 418 µL of distilled water and 187 µL of deuterated phosphate buffer containing 5.8 mM sodium 3-(trimethylsilyl)propionate-2,2,3,3-d_4_ (TSP) and NaN_3_. The pH was adjusted to 4.20 ± 0.02 with 8.5% H_3_PO_4_ or NaOH. Then, the solutions were transferred into 5 mm NMR tubes. The moisture content of each sample was measured refractometrically [19].

### 3.3. NMR Spectroscopy

^13^C NMR measurements were performed on a Bruker Avance Neo 600 spectrometer (Bruker BioSpin GmbH, Rheinstetten, Germany) operating at 150.9 MHz and 300.0 ± 0.1 K. Data were acquired using the *zgdc30* pulse sequence with a 30° pulse angle, a spectral width of 238 ppm, 64 K data points, 8192 scans, an acquisition time of 0.90 s, and a relaxation delay of 1.05 s. Free induction decays were processed using TopSpin 4.5.0. Prior to Fourier transformation, an exponential window function corresponding to a line broadening of 2 Hz was applied. The resulting spectra were manually phase- and baseline-corrected. Chemical shifts were referenced to the α-fructofuranose resonance at δ 104.34 ppm. Resonance assignments followed the previously established assignment scheme [19].

### 3.4. Semiquantitative Analysis

Semiquantitative analysis was performed as previously described [19]. In brief, one non-overlapping CH resonance was selected for each constituent in the δ ^13^C 106–67 ppm region, and its peak height was used for semiquantitative analysis. The signal heights were converted to relative mass contributions by accounting for the corresponding molecular mass and, when applicable, the relative abundance of the quantified tautomer, as determined from the ^13^C NMR spectra of authentic compounds in D_2_O. Sixteen reproducible unassigned resonances were designated U1–U16 and estimated using an approximate molecular mass of 200 g/mol, based on previously obtained DOSY NMR data. The calculated contributions were normalized to the total and expressed as g/100 g honey, accounting for the moisture content used for the corresponding sample. When no quantifiable resonance was observed, the corresponding value was set to zero for the descriptive and statistical analyses [44].

### 3.5. Statistical Analysis

Descriptive statistics were calculated for the entire dataset and for each geographical group. Since several variables showed skewed distributions, contained numerous zero values, and exhibited non-linear relationships, the associations between the quantified constituents and reproducible unidentified signals were evaluated using Spearman rank correlation coefficients. The resulting 210 *p*-values for the 21 quantified saccharides were adjusted using the Benjamini–Hochberg false-discovery-rate procedure, and adjusted *p*-values below 0.05 were considered statistically significant. Associations involving the non-saccharide constituents and reproducible unidentified signals were examined descriptively.

Country-level discrimination was evaluated using linear discriminant analysis (LDA) in PAST version 4.10. LDA was selected because it provides a direct multiclass classification framework for the eight predefined country groups. OPLS-DA was used for the binary regional comparison between Strandzha PDO and honeys from other Bulgarian regions. Before analysis, the variables were standardized to z-scores. An initial model containing all variables was reduced iteratively by backward elimination. Variables were retained when removing them decreased the overall jackknife accuracy or impaired the classification of any geographical group. We assessed the performance of the final model using reclassification and leave-one-out jackknife cross-validation.

An exploratory CHAID classification tree was constructed in SIPINA version 3.13 using the complete dataset and the default settings to identify concentration thresholds that can be interpreted chemically. The reported accuracy represents apparent fit rather than independent predictive validation. The 95% confidence intervals for the LDA and decision tree accuracy estimates were calculated using the Wilson score method.

The 29 Bulgarian samples were analyzed separately using HCA and OPLS-DA in SIMCA 18.0 (Sartorius Stedim Data Analytics AB, Umeå, Sweden). For the SIMCA analyses, the variables were mean-centered and scaled to unit variance, and the default seven-fold cross-validation procedure was used for OPLS-DA. HCA was performed using Ward’s method and included all quantified constituents and reproducible unidentified signals. OPLS-DA was used to compare 19 Strandzha PDO samples with 10 samples from other Bulgarian regions. Variables with VIP values above 0.9 were retained from an initial model containing the complete variable set. The quality of the model was described by R^2^X, R^2^Y, and Q^2^ values. The model was evaluated using cross-validated classification, CV-ANOVA, and receiver operating characteristic (ROC) analysis. A 200-permutation test was also performed, in which the class labels were randomly permuted and the R^2^Y and Q^2^ regression-line intercepts at zero correlation were calculated.

## 4. Conclusions

A semiquantitative ^13^C NMR profiling analysis of 60 oak honeydew honeys from eight European countries revealed geographical differentiation at two spatial scales. At the country level, LDA and decision tree analysis showed that national groups were resolved through combinations of saccharide concentrations and minor constituents detectable by NMR, rather than through a single universal discriminator. Within Bulgaria, hierarchical cluster analysis and orthogonal partial least squares discriminant analysis used the same extended molecular profile to distinguish Strandzha PDO honeys from honeys originating from other regions. These results demonstrate that geographical information is retained at both the national and subnational levels.

The chemical analysis revealed an organized structure among the minor saccharides. Turanose, maltulose, trehalulose, isomaltose, kojibiose, nigerose, and α,β-trehalose exhibited the strongest correlations, while α,α-trehalose was associated with a looser group of variably occurring trisaccharides. These relationships were not geographically specific. Instead, differences in the relative expression of their individual members explain why classification models rely on different combinations of compounds across countries and regions. The reproducible presence of several unidentified NMR signals suggests that the chemical basis of geographical differentiation remains partially unresolved.

Previously proposed compounds as indicators of honeydew or oak origin yielded inconsistent results. Melezitose and 2,3-butanediol were either absent or highly variable in some parts of the dataset, therefore they could not be considered universal components of European oak honeydew honey. In contrast, 1-kestose was quantified in every sample from all eight countries and emerged as the most consistent indicator candidate. However, its specificity to oak-derived honeydew remains to be determined.

Overall, the results support the use of extended semiquantitative ^13^C NMR profiling for the exploratory geographical characterization of European oak honeydew honey and the development of regional authentication approaches. However, validation in larger, independent datasets—including blossom and non-oak honeydew controls—and structural identification of the unidentified signals are necessary before the proposed indicators and classification models can be used as standalone authentication tools.

## Figures and Tables

**Table 1:** ^13^C NMR chemical shifts and descriptive statistics for the saccharides assigned in European oak honeydew honeys.

Saccharide	Abbreviation	δC, ppm	Frequency (n/60)	Range	Mean	Median
*Monosaccharides*						
Fructose	F	67.54	60/60	24.36–38.96	32.82	33.17
Glucose	G	74.10	60/60	23.21–40.63	32.06	31.59
*Disaccharides*						
Gentiobiose	Gb	102.45	31/60	0.00–0.46	0.10	0.06
Sucrose	Su	76.36	58/60	0.00–1.04	0.29	0.24
Isomaltose	IMa	97.73	60/60	0.71–4.02	1.62	1.50
Isomaltulose	IMu	104.65	44/60	0.00–0.92	0.34	0.31
Kojibiose	Kb	89.30	60/60	0.43–1.41	0.87	0.84
Leucrose	Lu	100.04	59/60	0.00–2.70	0.36	0.22
Maltose	Ma	99.48	60/60	0.35–2.57	1.03	0.99
Maltulose	Mu	100.28	58/60	0.00–5.49	2.11	1.96
Nigerose	Ng	98.80	60/60	0.22–1.20	0.57	0.54
α,α-Trehalose	ααTr	93.00	46/60	0.00–4.58	0.49	0.12
α,β-Trehalose	αβTr	102.69	60/60	0.01–0.71	0.36	0.34
Trehalulose	Tru	97.69	60/60	0.54–3.58	1.35	1.17
Turanose	Tu	100.65	60/60	1.06–2.70	1.82	1.78
*Trisaccharides*						
Erlose	Er	99.59	58/60	0.00–2.54	0.45	0.27
1-Kestose	1-Ks	92.28	60/60	0.12–1.17	0.49	0.49
Maltotriose	Mr	99.41	35/60	0.00–1.12	0.22	0.04
Melezitose	Mz	83.36	33/60	0.00–3.74	0.30	0.04
Panose	Pa	99.56	54/60	0.00–3.87	0.45	0.35
Raffinose	Rf	76.24	60/60	0.04–0.90	0.35	0.34

Concentrations are expressed in g/100 g. Selected non-overlapping carbon–hydrogen (CH) resonances are reported. Frequency indicates the number of samples with a quantifiable resonance.

## Data Availability

The data presented in this study are available within the article and its Supporting Information. Additional data are available from the corresponding author upon reasonable request.

## Supplementary Materials

The following supporting information can be downloaded at: https://www.mdpi.com/article/10.3390/molecules31162913/s1, Figure S1: ^13^C NMR spectra (anomeric part) of selected European oak honeydew honeys from Spain (ES4), Serbia (RS8), Italy (IT1), and Bulgaria (BG2), showing selected resonances used for profiling isomaltose (IMa), isomaltulose (IMu), kojibiose (Kb), leucrose (Lu), maltose (Ma), maltulose (Mu), nigerose (Ng), α,α-trehalose (ααTr), α,β-trehalose (αβTr), trehalulose (Tru), turanose (Tu), erlose (Er), 1-kestose (1-Ks), melezitose (Mz), panose (Pa), and unidentified signal U6; Figure S2: Receiver operating characteristic curves based on cross-validated predicted class values for the regional OPLS-DA model. The class-specific curves overlap (AUC = 1.00 for each class); Figure S3: Results of the 200-permutation test for the regional OPLS-DA model; Table S1: Geographical origin, classification and collection year of the honey samples included in the study; Table S2: Country-specific mean concentrations and ranges of the assigned constituents and unidentified signals; Table S3: Loadings of the sixteen variables retained in the country-level LDA model across the seven discriminant functions; Table S4: Confusion matrices for the original and leave-one-out cross-validated country-level LDA classifications; Table S5: Confusion matrix for the OPLS-DA model differentiating Strandzha PDO from other Bulgarian honeys; Table S6: Spearman rank correlation coefficients among the 21 quantified saccharides; Table S7: Geographical origins declared by the producers and measured quercitol concentrations for the 15 samples excluded from this study.

## Abbreviations

The following abbreviations are used in this manuscript:

AUC	Area under the curve
CHAID	Chi-square automatic interaction detection
CV-ANOVA	Cross-validated analysis of variance
DOSY	Diffusion-ordered spectroscopy
HCA	Hierarchical cluster analysis
LC-MS/MS	Liquid chromatography–tandem mass spectrometry
LD	Linear discriminant function
LDA	Linear discriminant analysis
NMR	Nuclear magnetic resonance
OPLS-DA	Orthogonal partial least squares discriminant analysis
Other_BG	Oak honeydew honeys from Bulgarian regions outside the Strandzha PDO production area
PDO	Protected Designation of Origin
PLS-DA	Partial least squares discriminant analysis
Q^2^	Cross-validated predictive ability
R^2^	Goodness-of-fit parameter
R^2^X	Proportion of X-variance explained by the model
R^2^Y	Proportion of Y-variance explained by the model
ROC	Receiver operating characteristic
TSP	Sodium 3-(trimethylsilyl)propionate-2,2,3,3-d_4_
U1–U16	Reproducible unidentified ^13^C NMR signals 1–16
VIP	Variable importance in projection
