# Supplementary material for: NMR Profiling of European Oak Honeydew Honeys: Saccharide Diversity and Geographical Differentiation
Source: Molecules. 2026 Aug 20;31(16):2913. doi: 10.3390/molecules31162913 (PMC13515498; doi:10.3390/molecules31162913)
Supplement: Supplementary file 1 [file molecules-31-02913-s001.zip › molecules-4515214-supplementary.pdf]

NMR Profiling of European Oak Honeydew Honeys: Saccharide Diversity and Geographical Differentiation

Dessislava Gerginova[1,2], María del Carmen Seijo Coello[3], Nikola Jovanović[4], Marco Ciulu[5] and Svetlana Simova[1,2,*]

[1] Bulgarian NMR Centre, Institute of Organic Chemistry with Centre of Phytochemistry, Bulgarian Academy of Sciences, Acad. G. Bonchev Str., Bl. 9, 1113 Sofia, Bulgaria; Dessislava.Gerginova@orgchm.bas.bg (D.G.)
[2] Centre of Competence "Sustainable Utilization of Bio-Resources and Waste of Medicinal and Aromatic Plants for Innovative Bioactive Products" (BIORESOURCES BG), 1000 Sofia, Bulgaria
[3] Department of Plant Biology and Soil Science, Faculty of Sciences, University of Vigo, As Lagoas, 32004 Ourense, Spain; mcoello@uvigo.gal (M.C.S.C.)
[4] Department of Biology and Ecology, Faculty of Sciences and Mathematics, University of Niš, Višegradska 33, 18000 Niš, Serbia; nikola.jovanovic1@pmf.edu.rs (N.J.)
[5] Department of Biotechnology, University of Verona, Strada Le Grazie 15, 37134 Verona, Italy; marco.ciulu@univr.it (M.C.)
* Correspondence: Svetlana.Simova@orgchm.bas.bg (S.S.)

Figure S1. ^{13}C NMR spectra (anomeric part) of selected European oak honeydew honeys from Spain (ES4), Serbia (RS8), Italy (IT1), and Bulgaria (BG2), showing selected resonances used for profiling isomaltose (IMa), isomaltulose (IMu), kojibiose (Kb), leucrose (Lu), maltose (Ma), maltulose (Mu), nigerose (Ng), α,α-trehalose ($\alpha\alpha$Tr), α,β-trehalose (αβTr), trehalulose (Tru), turanose (Tu), erlose (Er), 1-kestose (1-Ks), melezitose (Mz), panose (Pa), and unidentified signal U6.

Figure S2. Receiver operating characteristic curves based on cross-validated predicted class values for the regional OPLS-DA model. The class-specific curves overlap (AUC = 1.00 for each class).

Figure S3. Results of the 200-permutation test for the regional OPLS-DA model.

Table S1. Geographical origin, classification and collection year of the honey samples included in the study.

Table S2. Country-specific mean concentrations and ranges of the assigned constituents and unidentified signals.

Table S3. Loadings of the sixteen variables retained in the country-level LDA model across the seven discriminant functions.

Table S4. Confusion matrices for the original and leave-one-out cross-validated country-level LDA classifications.

Table S5. Confusion matrix for the OPLS-DA model differentiating Strandzha PDO from other Bulgarian honeys.

Table S6. Spearman rank correlation coefficients among the 21 quantified saccharides.

Table S7. Geographical origins declared by the producers and measured quercitol concentrations for the 15 samples excluded from this study.

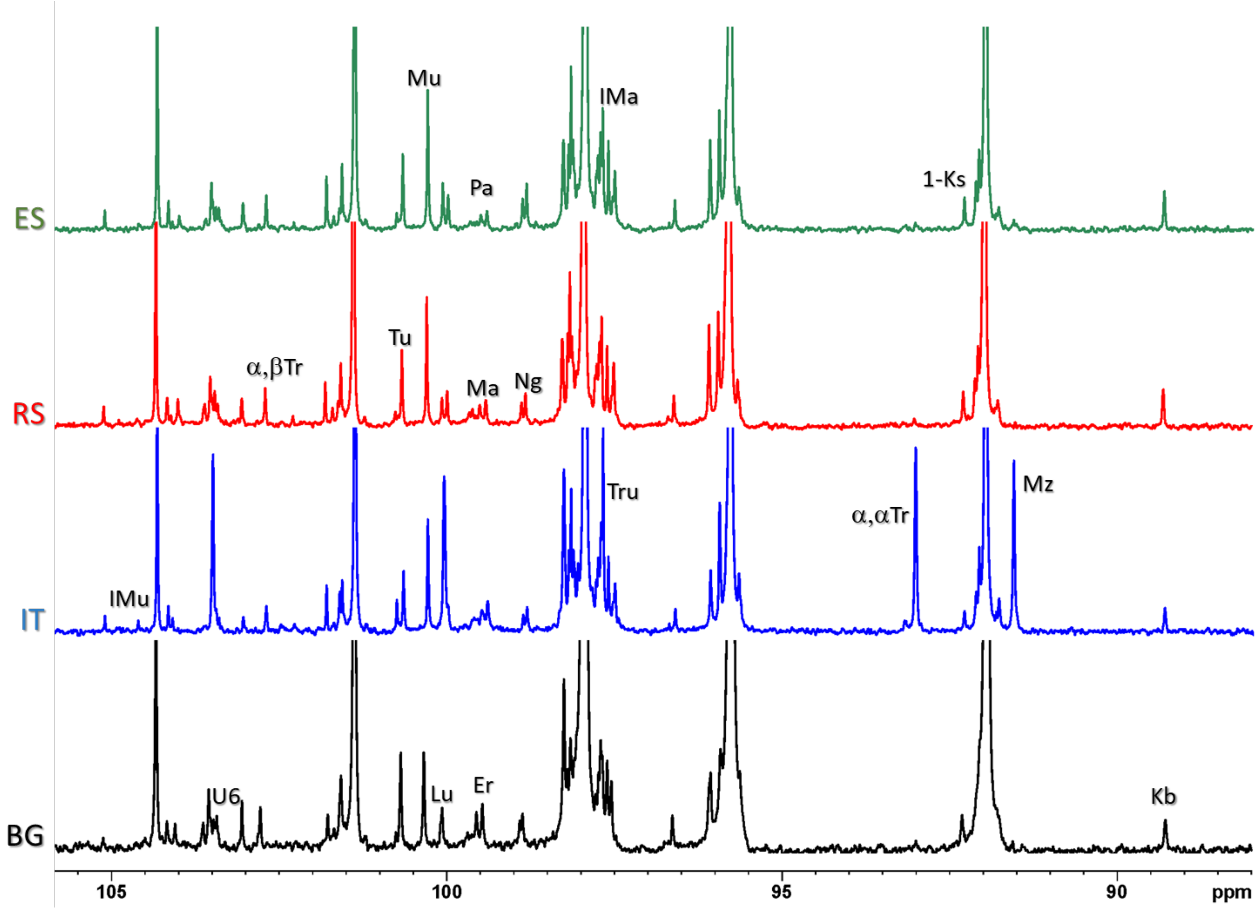

Figure S1. ^{13}C NMR spectra (anomeric part) of selected European oak honeydew honeys from Spain (ES4), Serbia (RS8), Italy (IT1), and Bulgaria (BG2), showing selected resonances used for profiling isomaltose (IMa), isomaltulose (IMu), kojibiose (Kb), leucrose (Lu), maltose (Ma), maltulose (Mu), nigerose (Ng), *α*,*α*-trehalose (*αα*Tr), *α*,β-trehalose (*α*βTr), trehalulose (Tru), turanose (Tu), erlose (Er), 1-kestose (1-Ks), melezitose (Mz), panose (Pa), and unidentified signal U6.

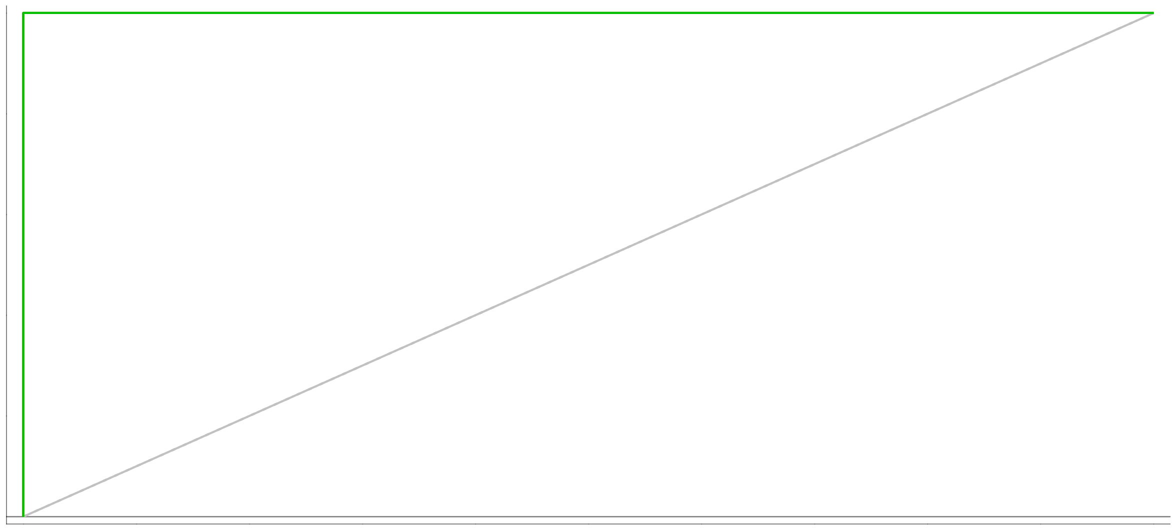

Figure S2. Receiver operating characteristic curves based on cross-validated predicted class values for the regional OPLS-DA model. The class-specific curves overlap (AUC = 1.00 for each class).

A) Other_BG

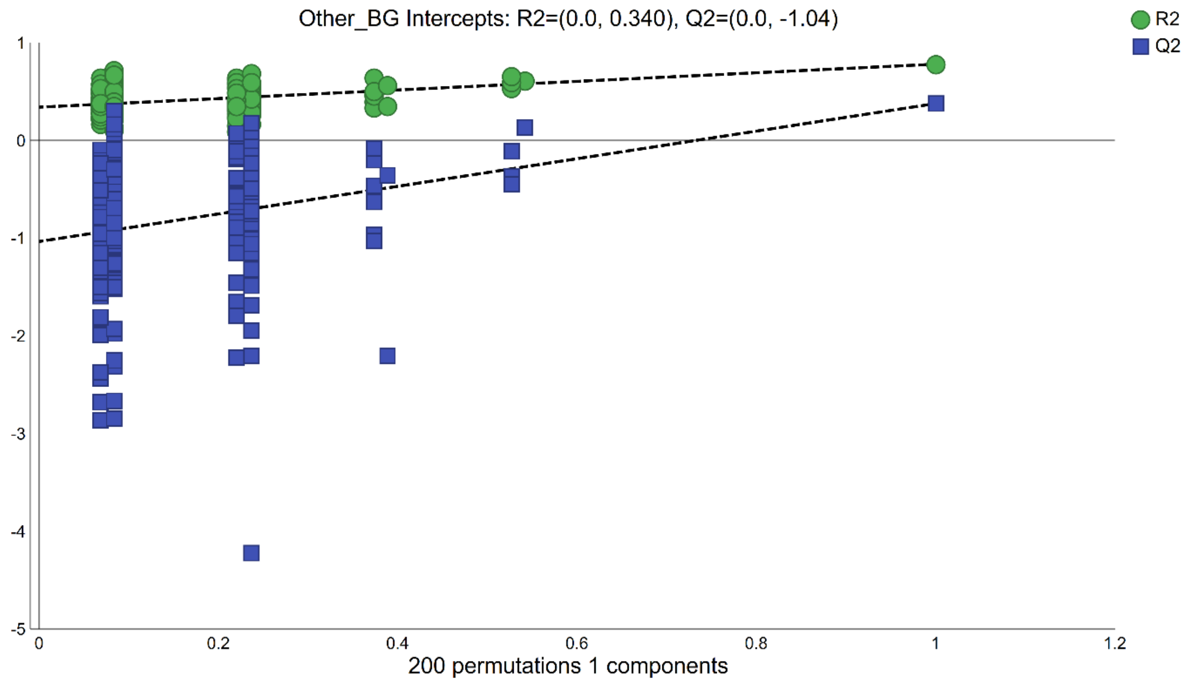

B) Strandzha

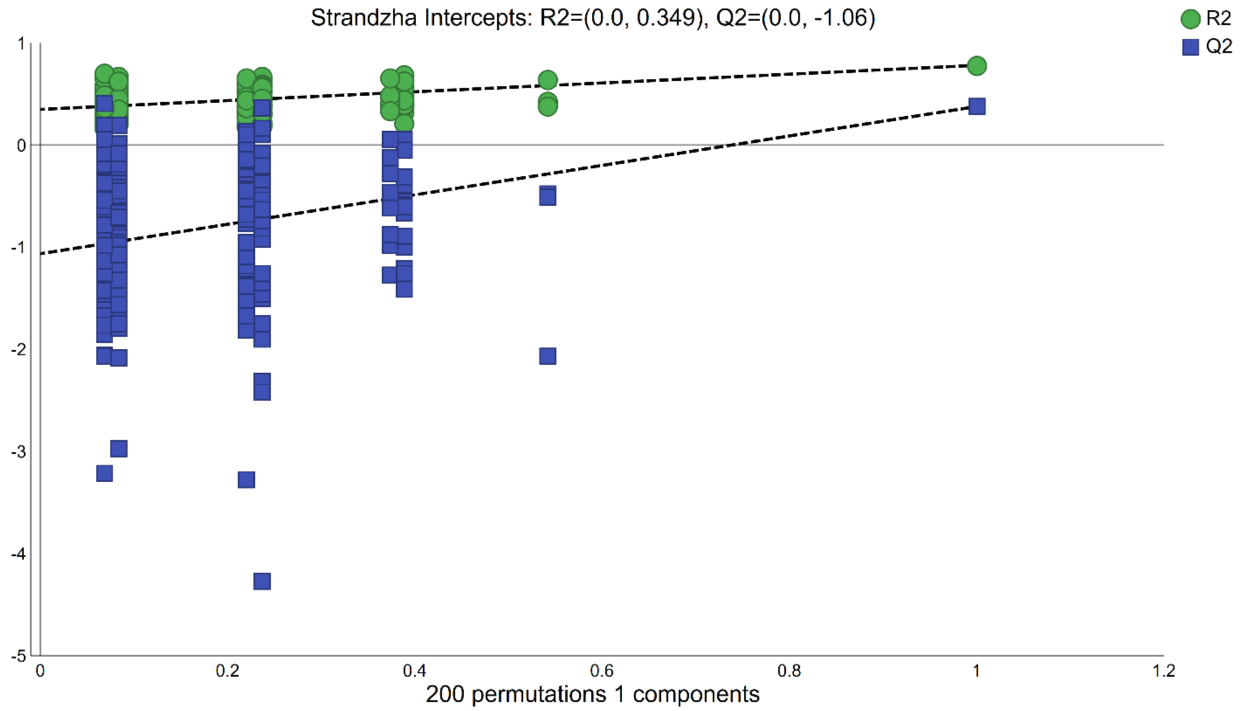

Figure S3. Results of the 200-permutation test for the regional OPLS-DA model.

Table S1. Geographical origin, classification and collection year of the honey samples included in the study.

Sample code	Country-level class	Declared geographical origin	Year	Bulgarian regional class
**S1**	BG	Strandzha PDO production area, Bulgaria	2016	Strandzha PDO
**S2**	BG	Strandzha PDO production area, Bulgaria	2016	Strandzha PDO
**S3**	BG	Strandzha PDO production area, Bulgaria	2016	Strandzha PDO
**S4**	BG	Strandzha PDO production area, Bulgaria	2019	Strandzha PDO
**S5**	BG	Strandzha PDO production area, Bulgaria	2019	Strandzha PDO
**S6**	BG	Strandzha PDO production area, Bulgaria	2016	Strandzha PDO
**S7**	BG	Strandzha PDO production area, Bulgaria	2020	Strandzha PDO
**S8**	BG	Strandzha PDO production area, Bulgaria	2020	Strandzha PDO
**S9**	BG	Strandzha PDO production area, Bulgaria	2021	Strandzha PDO
**S10**	BG	Strandzha PDO production area, Bulgaria	2016	Strandzha PDO
**S11**	BG	Strandzha PDO production area, Bulgaria	2016	Strandzha PDO
**S12**	BG	Strandzha PDO production area, Bulgaria	2016	Strandzha PDO
**S13**	BG	Strandzha PDO production area, Bulgaria	2016	Strandzha PDO
**S14**	BG	Strandzha PDO production area, Bulgaria	2025	Strandzha PDO
**S15**	BG	Strandzha PDO production area, Bulgaria	2025	Strandzha PDO
**S16**	BG	Strandzha PDO production area, Bulgaria	2025	Strandzha PDO
**S17**	BG	Strandzha PDO production area, Bulgaria	2025	Strandzha PDO
**S18**	BG	Strandzha PDO production area, Bulgaria	2025	Strandzha PDO
**S19**	BG	Strandzha PDO production area, Bulgaria	2025	Strandzha PDO
**BG1**	BG	Pazardzhik Province, Bulgaria	2016	Other_BG
**BG2**	BG	Sofia Province, Bulgaria	2016	Other_BG
**BG3**	BG	Varna Province, Bulgaria	2017	Other_BG
**BG4**	BG	Kyustendil Province, Bulgaria	2017	Other_BG
**BG5**	BG	Varna Province, Bulgaria	2016	Other_BG
**BG6**	BG	Plovdiv Province, Bulgaria	2016	Other_BG
**BG7**	BG	Elenski Balkan, Bulgaria	2025	Other_BG
**BG8**	BG	Stara Zagora Province, Bulgaria	2025	Other_BG
**BG9**	BG	Krumovgrad area, Kardzhali Province, Bulgaria	2025	Other_BG
**BG10**	BG	Vratsa Province, Bulgaria	2025	Other_BG
**DE1**	DE	Thuringian Forest, Germany	2017	—
**DE2**	DE	Thuringian Forest, Germany	2019	—
**GR1**	GR	Mount Athos, Greece	2016	—
**GR2**	GR	Halkidiki, Central Macedonia, Greece	2017	—
**GR3**	GR	Peloponnese Region, Greece	2019	—
**GR4**	GR	Thessaly Region, Greece	2020	—

**GR5**	GR	Thessaly Region, Greece	2020	—
**IT1**	IT	Trentino, Italy	2022	—
**IT2**	IT	Calabria, Italy	2022	—
**IT3**	IT	Apulia, Italy	2022	—
**NM1**	NM	Makedonski Brod Municipality, North Macedonia	2017	—
**NM2**	NM	Makedonski Brod Municipality, North Macedonia	2018	—
**NM3**	NM	Makedonski Brod Municipality, North Macedonia	2018	—
**RO1**	RO	Vâlcea County, Romania	2018	—
**RO2**	RO	Vâlcea County, Romania	2018	—
**RO3**	RO	Vâlcea County, Romania	2018	—
**RO4**	RO	Vâlcea County, Romania	2018	—
**RO5**	RO	Vâlcea County, Romania	2018	—
**RS1**	RS	Šumadija District, Serbia	2021	—
**RS2**	RS	Zlatibor District, Serbia	2021	—
**RS3**	RS	Moravica District, Serbia	2021	—
**RS4**	RS	Crni Vrh area, Serbia	2025	—
**RS5**	RS	Vlasina region, Serbia	2025	—
**RS6**	RS	Radan Mountain area, Serbia	2025	—
**RS7**	RS	Zaječar District, Serbia	2024	—
**RS8**	RS	Pirot District, Serbia	2024	—
**RS9**	RS	Blace area, Serbia	2021	—
**ES1**	ES	Asturias, Spain	2024	—
**ES2**	ES	Galicia, Spain	2024	—
**ES3**	ES	Andalusia, Spain	2024	—
**ES4**	ES	Galicia, Spain	2024	—

Country-level classes correspond to the eight geographical groups used in the LDA model. The Bulgarian regional class was used only for the Strandzha PDO versus Other_BG comparison.

Table S2. Country-specific mean concentrations and ranges of the assigned constituents and unidentified signals.

Constituent	Abbreviation	BG (n=29)	DE (n=2)	GR (n=5)	IT (n=3)	NM (n=3)	RO (n=5)	RS (n=9)	ES (n=4)
Fructose	F	33.60 (28.61–38.96)	31.20 (30.41–31.99)	33.56 (32.45–34.95)	27.95 (26.96–28.73)	33.55 (33.13–34.34)	37.58 (36.25–38.87)	30.26 (24.36–34.65)	30.04 (28.97–30.84)
Glucose	G	33.04 (27.05–40.20)	29.51 (28.24–30.78)	30.39 (27.74–31.81)	33.93 (31.06–36.69)	30.03 (29.08–31.73)	31.08 (28.97–32.19)	31.35 (23.21–40.63)	31.25 (28.13–33.40)
Gentiobiose	Gb	0.09 (0.00–0.28)	0.17 (0.17–0.17)	0.11 (0.00–0.21)	0.19 (0.00–0.30)	0.00 (0.00–0.00)	0.00 (0.00–0.00)	0.08 (0.00–0.18)	0.28 (0.17–0.46)
Sucrose	Su	0.27 (0.00–1.04)	0.29 (0.21–0.36)	0.65 (0.17–0.95)	0.25 (0.14–0.43)	0.18 (0.12–0.26)	0.15 (0.09–0.30)	0.25 (0.05–0.89)	0.30 (0.09–0.43)
Isomaltose	IMa	1.47 (0.83–2.88)	1.65 (1.53–1.76)	1.31 (0.71–2.00)	2.69 (1.84–4.02)	2.02 (1.67–2.26)	1.14 (0.81–1.87)	1.53 (0.77–2.55)	2.81 (2.13–3.33)
Isomaltulose	IMu	0.29 (0.00–0.74)	0.14 (0.00–0.29)	0.39 (0.00–0.88)	0.80 (0.73–0.92)	0.51 (0.27–0.66)	0.20 (0.00–0.48)	0.33 (0.00–0.82)	0.43 (0.00–0.72)
Kojibiose	Kb	0.80 (0.43–1.41)	0.54 (0.43–0.66)	0.76 (0.58–1.05)	1.06 (0.76–1.25)	1.21 (0.98–1.37)	0.73 (0.63–0.93)	0.90 (0.63–1.35)	1.31 (1.19–1.38)
Leucrose	Lu	0.23 (0.14–0.87)	1.44 (0.18–2.70)	0.18 (0.10–0.27)	0.31 (0.00–0.49)	0.32 (0.30–0.34)	0.18 (0.04–0.42)	0.57 (0.14–2.13)	0.83 (0.67–1.11)
Maltose	Ma	0.98 (0.42–1.99)	1.03 (0.84–1.23)	1.79 (0.98–2.37)	0.63 (0.52–0.69)	0.44 (0.38–0.48)	0.70 (0.35–1.26)	1.45 (0.67–2.57)	0.59 (0.49–0.88)
Maltulose	Mu	2.00 (1.24–4.27)	1.14 (1.05–1.22)	1.54 (0.94–2.20)	3.15 (2.69–3.82)	2.40 (1.78–2.80)	1.76 (1.19–2.24)	1.60 (0.00–3.08)	4.68 (3.61–5.49)
Nigerose	Ng	0.55 (0.28–0.84)	0.44 (0.44–0.45)	0.45 (0.36–0.59)	0.80 (0.56–0.93)	0.61 (0.45–0.69)	0.36 (0.22–0.51)	0.51 (0.32–0.70)	1.13 (1.03–1.20)
α,α-Trehalose	ααTr	0.20 (0.00–1.88)	1.67 (1.36–1.97)	0.14 (0.00–0.40)	0.93 (0.00–2.37)	0.12 (0.09–0.17)	0.07 (0.00–0.37)	1.77 (0.00–4.58)	0.10 (0.00–0.23)
α,β-Trehalose	αβTr	0.37 (0.21–0.53)	0.29 (0.28–0.31)	0.31 (0.21–0.39)	0.43 (0.34–0.49)	0.41 (0.31–0.47)	0.23 (0.16–0.33)	0.30 (0.01–0.48)	0.59 (0.48–0.71)
Trehalulose	Tru	1.12 (0.54–1.72)	1.21 (0.76–1.67)	0.93 (0.63–1.33)	2.74 (1.99–3.58)	1.38 (0.95–1.65)	1.02 (0.77–1.64)	1.71 (0.84–2.90)	2.19 (1.69–2.77)
Turanose	Tu	1.86 (1.40–2.35)	1.24 (1.06–1.42)	1.68 (1.47–2.03)	2.20 (1.77–2.70)	1.81 (1.65–1.92)	1.49 (1.18–1.86)	1.66 (1.41–2.21)	2.48 (2.37–2.63)
Erlose	Er	0.33 (0.00–1.06)	0.41 (0.31–0.50)	1.04 (0.41–1.52)	0.25 (0.00–0.47)	0.16 (0.11–0.25)	0.22 (0.06–0.48)	0.90 (0.17–2.54)	0.18 (0.13–0.27)
1-Kestose	1-Ks	0.51 (0.17–1.17)	0.78 (0.67–0.90)	0.53 (0.31–0.69)	0.46 (0.21–0.76)	0.52 (0.49–0.54)	0.30 (0.13–0.57)	0.41 (0.12–0.78)	0.51 (0.14–0.70)
Maltotriose	Mr	0.14 (0.00–0.56)	0.25 (0.00–0.50)	0.46 (0.00–0.80)	0.21 (0.00–0.62)	0.00 (0.00–0.00)	0.01 (0.00–0.05)	0.57 (0.00–1.12)	0.08 (0.05–0.14)
Melezitose	Mz	0.13 (0.00–1.38)	3.13 (2.52–3.74)	0.02 (0.00–0.09)	1.13 (0.00–3.40)	0.00 (0.00–0.00)	0.05 (0.00–0.14)	0.39 (0.00–1.98)	0.22 (0.00–0.62)
Panose	Pa	0.27 (0.00–0.55)	0.60 (0.50–0.69)	0.67 (0.33–1.07)	0.41 (0.00–0.82)	0.36 (0.35–0.38)	0.12 (0.00–0.36)	1.13 (0.20–3.87)	0.37 (0.27–0.58)
Raffinose	Rf	0.36 (0.07–0.90)	0.45 (0.36–0.54)	0.41 (0.19–0.86)	0.26 (0.11–0.37)	0.55 (0.51–0.58)	0.35 (0.23–0.46)	0.31 (0.04–0.59)	0.20 (0.14–0.31)
Quercitol	Q	0.40 (0.22–0.84)	0.25 (0.25–0.25)	0.29 (0.23–0.47)	0.58 (0.48–0.76)	0.72 (0.69–0.77)	0.51 (0.25–0.83)	0.46 (0.24–0.82)	0.34 (0.28–0.38)
Proline	Pro	0.08 (0.04–0.18)	0.06 (0.06–0.06)	0.07 (0.04–0.13)	0.08 (0.07–0.10)	0.08 (0.07–0.10)	0.09 (0.08–0.10)	0.07 (0.04–0.11)	0.10 (0.08–0.12)
meso-2,3-Butanediol	mBd	0.11 (0.02–0.35)	0.01 (0.00–0.01)	0.09 (0.02–0.22)	0.06 (0.00–0.19)	0.10 (0.03–0.24)	0.12 (0.04–0.18)	0.09 (0.00–0.37)	0.09 (0.00–0.15)
rac-2,3-Butanediol	rBd	0.05 (0.01–0.15)	0.00 (0.00–0.00)	0.03 (0.00–0.08)	0.02 (0.00–0.07)	0.04 (0.01–0.10)	0.05 (0.02–0.10)	0.05 (0.00–0.20)	0.05 (0.04–0.07)
Unidentified signal U1	U1	0.08 (0.00–0.23)	0.03 (0.00–0.05)	0.04 (0.00–0.11)	0.11 (0.09–0.13)	0.13 (0.06–0.24)	0.02 (0.00–0.04)	0.09 (0.03–0.17)	0.08 (0.07–0.11)
Unidentified signal U2	U2	0.11 (0.00–0.27)	0.14 (0.09–0.20)	0.18 (0.12–0.38)	0.00 (0.00–0.00)	0.13 (0.00–0.34)	0.07 (0.03–0.10)	0.07 (0.00–0.16)	0.02 (0.00–0.10)
Unidentified signal U3	U3	0.14 (0.00–0.32)	0.00 (0.00–0.00)	0.29 (0.14–0.46)	0.00 (0.00–0.00)	0.10 (0.00–0.23)	0.03 (0.00–0.10)	0.36 (0.00–1.55)	0.00 (0.00–0.00)

Constituent	Abbreviation	BG (n=29)	DE (n=2)	GR (n=5)	IT (n=3)	NM (n=3)	RO (n=5)	RS (n=9)	ES (n=4)
Unidentified signal U4	U4	0.14 (0.00–0.31)	0.40 (0.36–0.44)	0.32 (0.00–0.62)	0.00 (0.00–0.00)	0.14 (0.00–0.22)	0.05 (0.00–0.13)	0.32 (0.08–1.05)	0.00 (0.00–0.00)
Unidentified signal U5	U5	0.13 (0.00–0.25)	0.29 (0.09–0.50)	0.07 (0.00–0.11)	0.15 (0.11–0.20)	0.19 (0.17–0.21)	0.09 (0.00–0.27)	0.15 (0.00–0.70)	0.15 (0.07–0.21)
Unidentified signal U6	U6	0.03 (0.00–0.08)	0.00 (0.00–0.00)	0.01 (0.00–0.03)	0.05 (0.03–0.06)	0.16 (0.10–0.19)	0.12 (0.08–0.17)	0.04 (0.00–0.10)	0.03 (0.02–0.04)
Unidentified signal U7	U7	0.02 (0.00–0.06)	0.12 (0.08–0.16)	0.03 (0.00–0.06)	0.02 (0.00–0.05)	0.02 (0.00–0.05)	0.00 (0.00–0.00)	0.04 (0.00–0.17)	0.03 (0.02–0.05)
Unidentified signal U8	U8	0.05 (0.02–0.08)	0.06 (0.05–0.06)	0.06 (0.05–0.07)	0.06 (0.05–0.07)	0.02 (0.00–0.07)	0.04 (0.00–0.06)	0.05 (0.00–0.08)	0.06 (0.06–0.06)
Unidentified signal U9	U9	0.09 (0.05–0.14)	0.08 (0.06–0.10)	0.08 (0.06–0.13)	0.10 (0.07–0.15)	0.11 (0.10–0.13)	0.08 (0.04–0.10)	0.08 (0.04–0.13)	0.09 (0.07–0.11)
Unidentified signal U10	U10	0.08 (0.05–0.13)	0.09 (0.05–0.12)	0.04 (0.00–0.09)	0.07 (0.06–0.08)	0.08 (0.05–0.12)	0.07 (0.05–0.09)	0.07 (0.05–0.08)	0.08 (0.07–0.08)
Unidentified signal U11	U11	0.11 (0.06–0.20)	0.11 (0.10–0.12)	0.09 (0.05–0.13)	0.24 (0.17–0.30)	0.14 (0.12–0.18)	0.10 (0.06–0.14)	0.15 (0.06–0.27)	0.17 (0.14–0.21)
Unidentified signal U12	U12	0.00 (0.00–0.06)	0.00 (0.00–0.00)	0.00 (0.00–0.00)	0.00 (0.00–0.00)	0.01 (0.00–0.04)	0.00 (0.00–0.00)	0.02 (0.00–0.04)	0.01 (0.00–0.03)
Unidentified signal U13	U13	0.69 (0.38–1.59)	0.39 (0.38–0.41)	0.60 (0.46–0.80)	1.25 (1.08–1.47)	0.74 (0.71–0.76)	0.50 (0.45–0.55)	0.83 (0.47–1.13)	1.21 (0.00–1.93)
Unidentified signal U14	U14	0.37 (0.02–0.78)	0.33 (0.27–0.40)	0.34 (0.24–0.52)	0.43 (0.31–0.58)	0.36 (0.29–0.47)	0.29 (0.21–0.33)	0.36 (0.31–0.42)	0.60 (0.46–0.92)
Unidentified signal U15	U15	0.06 (0.00–0.10)	0.05 (0.05–0.05)	0.05 (0.00–0.08)	0.04 (0.00–0.07)	0.05 (0.05–0.06)	0.04 (0.00–0.06)	0.05 (0.00–0.08)	0.07 (0.06–0.09)
Unidentified signal U16	U16	0.01 (0.00–0.06)	0.00 (0.00–0.00)	0.01 (0.00–0.03)	0.01 (0.00–0.02)	0.07 (0.05–0.11)	0.00 (0.00–0.02)	0.01 (0.00–0.05)	0.00 (0.00–0.00)
Sum of U1–U16	ΣU1–U16	2.11 (1.48–3.00)	2.09 (1.92–2.27)	2.20 (1.89–2.54)	2.53 (2.27–2.88)	2.46 (2.22–2.82)	1.50 (1.42–1.69)	2.71 (1.76–4.17)	2.62 (1.91–3.22)

Values are presented as mean (min–max), g/100 g honey. Country codes: BG, Bulgaria; DE, Germany; GR, Greece; IT, Italy; NM, North Macedonia; RO, Romania; RS, Serbia; ES, Spain. ΣU1–U16 is the summed contribution of the sixteen reproducible unidentified signals.

Table S3. Loadings of the sixteen variables retained in the country-level LDA model across the seven discriminant functions.

Variable	LD1	LD2	LD3	LD4	LD5	LD6	LD7
Glucose (G)	0.11814	-2.0025	-1.2872	1.0755	1.9513	-0.55877	-1.3942
Fructose (F)	-0.28329	-1.4172	-0.34334	0.97968	1.1426	-0.47505	-2.2021
Kojibiose (Kb)	1.2596	0.95171	-0.53286	-0.13873	-1.2579	1.7507	0.087329
Nigerose (Ng)	-0.89795	-2.9946	1.5830	-0.41475	-0.29435	-1.1420	-1.1147
α,α-Trehalose ($\alpha\alpha$Tr)	-0.44636	-1.4791	-1.0161	0.37128	-0.075131	-0.4887	-1.6395
α,β-Trehalose ($\alpha\beta$Tr)	-0.47261	0.70088	0.031141	0.30723	1.7794	0.0036015	-0.28547
Trehalulose (Tru)	-0.44298	-2.1146	0.39985	0.26776	-0.16768	-1.3904	0.014886
rac-2,3-butanediol (rBd)	-0.028875	-0.28451	-0.59371	0.44385	-0.094711	-0.49726	-0.33146
ΣU1–U16	-1.3869	0.18418	-2.9045	-2.0213	1.5739	-0.93332	-1.8319
U1	0.45703	-0.57808	0.39688	0.89308	-0.20703	0.76069	0.27913
U3	0.5224	-1.3677	1.615	2.0004	-0.87299	1.1649	0.75944
U6	2.0876	-0.59167	0.38665	0.26346	-0.24423	-0.62493	-0.053304
U11	0.78306	0.01371	-1.7531	0.12421	1.7734	0.5652	0.64322
U13	0.42547	-0.13283	2.2016	1.283	-1.2527	0.77966	0.98936
U14	0.15437	0.4719	1.1405	0.57747	-0.84459	0.46738	0.018321
U16	0.88301	-0.40513	0.019232	-0.72362	0.49611	0.11128	-0.11763

Table S4. Confusion matrices for the original and leave-one-out cross-validated country-level LDA classifications.

A) Original classification (60/60 correctly classified; 100.0%)

	BG	DE	GR	NM	RO	RS	ES	IT	Total
**BG**	29	0	0	0	0	0	0	0	29
**DE**	0	2	0	0	0	0	0	0	2
**GR**	0	0	5	0	0	0	0	0	5
**NM**	0	0	0	3	0	0	0	0	3
**RO**	0	0	0	0	5	0	0	0	5
**RS**	0	0	0	0	0	9	0	0	9
**ES**	0	0	0	0	0	0	4	0	4
**IT**	0	0	0	0	0	0	0	3	3
**Total**	29	2	5	3	5	9	4	3	60

B) Leave-one-out cross-validation (53/60 correctly classified; 88.3%)

	BG	DE	GR	NM	RO	RS	ES	IT	Total
**BG**	28	0	0	0	0	1	0	0	29
**DE**	0	2	0	0	0	0	0	0	2
**GR**	0	0	5	0	0	0	0	0	5
**NM**	0	0	0	3	0	0	0	0	3
**RO**	1	0	0	0	4	0	0	0	5
**RS**	2	1	0	0	0	6	0	0	9
**ES**	1	0	0	0	0	0	3	0	4
**IT**	0	0	0	0	0	1	0	2	3
**Total**	32	3	5	3	4	8	3	2	60

Rows represent declared countries; columns represent predicted countries.

Table S5. Confusion matrix for the OPLS-DA model differentiating Strandzha PDO from other Bulgarian honeys.

Actual class	Predicted Other_BG	Predicted Strandzha	Total
**Other_BG**	10	0	10
**Strandzha**	0	19	19
**Total**	10	19	29

Overall classification accuracy: 100%.

Table S6. Spearman rank correlation coefficients among the 21 quantified saccharides.

Saccharide	F	G	Gb	Su	IMa	IMu	Kb	Lu	Ma	Mu	Ng	ααTr	αβTr	Tru	Tu	Er	1-Ks	Mr	Mz	Pa	Rf
**Fructose**	—	**-0.37**	**-0.51**	-0.19	-0.22	-0.23	-0.28	**-0.40**	-0.18	**-0.36**	**-0.43**	**-0.38**	**-0.37**	**-0.33**	**-0.41**	**-0.34**	**-0.46**	**-0.40**	0.06	**-0.44**	**0.47**
**Glucose**	**-0.37**	—	0.22	-0.07	**-0.45**	-0.16	0.00	-0.27	-0.09	0.06	-0.09	-0.07	-0.03	-0.23	0.11	-0.03	0.05	-0.01	-0.16	**-0.34**	**-0.77**
**Gentiobiose**	**-0.51**	0.22	—	0.09	0.27	0.08	0.25	**0.32**	0.05	0.20	**0.33**	**0.35**	**0.33**	0.27	**0.32**	0.15	0.21	0.16	0.27	0.14	**-0.39**
**Sucrose**	-0.19	-0.07	0.09	—	0.02	0.03	0.06	-0.06	**0.31**	-0.05	0.29	-0.13	0.29	-0.04	0.28	0.13	**0.61**	-0.03	-0.13	0.24	0.01
**Isomaltose**	-0.22	**-0.45**	0.27	0.02	—	**0.42**	**0.54**	**0.55**	-0.28	**0.55**	**0.72**	0.24	**0.65**	**0.75**	**0.53**	-0.26	0.17	-0.12	0.03	0.26	**0.40**
**Isomaltulose**	-0.23	-0.16	0.08	0.03	**0.42**	—	0.27	0.18	-0.25	**0.39**	**0.33**	-0.01	0.19	**0.48**	0.26	-0.08	0.01	0.08	-0.25	0.04	0.10
**Kojibiose**	-0.28	0.00	0.25	0.06	**0.54**	0.27	—	**0.45**	**-0.32**	**0.47**	**0.72**	0.09	**0.74**	**0.68**	**0.60**	-0.22	0.17	-0.15	-0.19	-0.01	-0.13
**Leucrose**	**-0.40**	-0.27	**0.32**	-0.06	**0.55**	0.18	**0.45**	—	-0.11	**0.40**	**0.46**	**0.32**	**0.46**	**0.50**	**0.31**	-0.04	0.17	0.13	0.06	0.27	0.01
**Maltose**	-0.18	-0.09	0.05	**0.31**	-0.28	-0.25	**-0.32**	-0.11	—	**-0.46**	-0.17	0.20	-0.09	**-0.40**	-0.19	**0.72**	0.18	**0.41**	0.10	**0.51**	0.02
**Maltulose**	**-0.36**	0.06	0.20	-0.05	**0.55**	**0.39**	**0.47**	**0.40**	**-0.46**	—	**0.66**	-0.06	**0.55**	**0.47**	**0.67**	**-0.33**	0.06	-0.15	-0.25	-0.12	-0.08
**Nigerose**	**-0.43**	-0.09	**0.33**	0.29	**0.72**	**0.33**	**0.72**	**0.46**	-0.17	**0.66**	—	0.04	**0.91**	**0.61**	**0.83**	-0.22	**0.35**	-0.08	-0.16	0.14	0.03
**α,α-Trehalose**	**-0.38**	-0.07	**0.35**	-0.13	0.24	-0.01	0.09	**0.32**	0.20	-0.06	0.04	—	0.03	0.25	-0.16	**0.34**	0.09	0.28	**0.41**	**0.45**	-0.04
**α,β-Trehalose**	**-0.37**	-0.03	**0.33**	0.29	**0.65**	0.19	**0.74**	**0.46**	-0.09	**0.55**	**0.91**	0.03	—	**0.45**	**0.83**	-0.24	**0.37**	-0.19	-0.22	0.03	0.00
**Trehalulose**	**-0.33**	-0.23	0.27	-0.04	**0.75**	**0.48**	**0.68**	**0.50**	**-0.40**	**0.47**	**0.61**	0.25	**0.45**	—	**0.40**	-0.20	0.10	0.06	0.09	0.18	0.04
**Turanose**	**-0.41**	0.11	**0.32**	0.28	**0.53**	0.26	**0.60**	**0.31**	-0.19	**0.67**	**0.83**	-0.16	**0.83**	**0.40**	—	-0.23	**0.37**	-0.18	**-0.32**	-0.04	-0.13
**Erlose**	**-0.34**	-0.03	0.15	0.13	-0.26	-0.08	-0.22	-0.04	**0.72**	**-0.33**	-0.22	**0.34**	-0.24	-0.20	-0.23	—	0.11	**0.55**	0.18	**0.57**	-0.13
**1-Kestose**	**-0.46**	0.05	0.21	**0.61**	0.17	0.01	0.17	0.17	0.18	0.06	**0.35**	0.09	**0.37**	0.10	**0.37**	0.11	—	0.00	-0.18	**0.31**	-0.12
**Maltotriose**	**-0.40**	-0.01	0.16	-0.03	-0.12	0.08	-0.15	0.13	**0.41**	-0.15	-0.08	0.28	-0.19	0.06	-0.18	**0.55**	0.00	—	0.16	**0.53**	-0.19
**Melezitose**	0.06	-0.16	0.27	-0.13	0.03	-0.25	-0.19	0.06	0.10	-0.25	-0.16	**0.41**	-0.22	0.09	**-0.32**	0.18	-0.18	0.16	—	0.15	0.02
**Panose**	**-0.44**	**-0.34**	0.14	0.24	0.26	0.04	-0.01	0.27	**0.51**	-0.12	0.14	**0.45**	0.03	0.18	-0.04	**0.57**	**0.31**	**0.53**	0.15	—	0.18
**Raffinose**	**0.47**	**-0.77**	**-0.39**	0.01	**0.40**	0.10	-0.13	0.01	0.02	-0.08	0.03	-0.04	0.00	0.04	-0.13	-0.13	-0.12	-0.19	0.02	0.18	—

Values in bold indicate statistically significant correlations after Benjamini–Hochberg correction for 210 pairwise comparisons (adjusted $p < 0.05$). Abbreviations are defined in Table 1 of the main manuscript.

Table S7. Geographical origins declared by the producers and measured quercitol concentrations for the 15 samples excluded from this study.

Excluded sample №	Declared geographical origin	Quercitol (g/100 g)
**1**	Braničevo District, Serbia	0.00
**2**	Pirot District, Serbia	0.00
**3**	Vidin Province, Bulgaria	0.18
**4**	Marche, Italy	0.11
**5**	Apulia, Italy	0.00
**6**	Emilia-Romagna, Italy	0.00
**7**	Primorje-Gorski Kotar County, Croatia	0.02
**8**	Zagreb County, Croatia	0.02
**9**	Koprivnica-Križevci County, Croatia	0.04
**10**	Debrca Municipality, North Macedonia	0.06
**11**	Kriva Palanka Municipality, North Macedonia	0.10
**12**	Extremadura, Spain	0.09
**13**	Vâlcea County, Romania	0.10
**14**	Crete Region, Greece	0.00
**15**	Black Forest, Germany	0.00
